# Synaptojanin2 Mutation Causes Progressive High-frequency Hearing Loss in Mice

**DOI:** 10.3389/fncel.2020.561857

**Published:** 2020-09-25

**Authors:** Elisa Martelletti, Neil J. Ingham, Oliver Houston, Johanna C. Pass, Jing Chen, Walter Marcotti, Karen P. Steel

**Affiliations:** ^1^Wolfson Centre for Age-Related Diseases, King’s College London, London, United Kingdom; ^2^Department of Biomedical Sciences, University of Sheffield, Sheffield, United Kingdom; ^3^Neuroscience Institute, University of Sheffield, Sheffield, United Kingdom

**Keywords:** synaptojanin2, mouse mutant, progressive hearing loss, hair cells, single hair cell recording, auditory function, exocytosis

## Abstract

Progressive hearing loss is very common in the human population but we know little about the underlying molecular mechanisms. Synaptojanin2 (*Synj2*) has been reported to be involved, as a mouse mutation led to a progressive increase in auditory thresholds with age. Synaptojanin2 is a phosphatidylinositol (PI) phosphatase that removes the five-position phosphates from phosphoinositides, such as PIP_2_ and PIP_3_, and is a key enzyme in clathrin-mediated endocytosis. To investigate the mechanisms underlying progressive hearing loss, we have studied a different mutation of mouse *Synj2* to look for any evidence of involvement of vesicle trafficking particularly affecting the synapses of sensory hair cells. Auditory brainstem responses (ABR) developed normally at first but started to decline between 3 and 4 weeks of age in *Synj2^tm1b^* mutants. At 6 weeks old, some evidence of outer hair cell (OHC) stereocilia fusion and degeneration was observed, but this was only seen in the extreme basal turn so cannot explain the raised ABR thresholds that correspond to more apical regions of the cochlear duct. We found no evidence of any defect in inner hair cell (IHC) exocytosis or endocytosis using single hair cell recordings, nor any sign of hair cell synaptic abnormalities. Endocochlear potentials (EP) were normal. The mechanism underlying progressive hearing loss in these mutants remains elusive, but our findings of raised distortion product otoacoustic emission (DPOAE) thresholds and signs of OHC degeneration both suggest an OHC origin for the hearing loss. Synaptojanin2 is not required for normal development of hearing but it is important for its maintenance.

## Introduction

Hearing impairment is the most common sensory loss in people at any age and in the UK more than 70% of people over 70 years old and 40% of over 50 years old have age-related hearing loss (ARHL, or presbyacusis^1^). Hearing loss can lead to social isolation and depression, and it is a risk factor associated with dementia (Gurgel et al., 2014) and with cognitive decline (Lin et al., 2013).

ARHL is commonly considered to be inevitable with aging; however, not all people are affected. Heritability studies suggest that variability between people has both genetic and environmental contributions (Gates et al., 1999; Destefano et al., 2003; Demeester et al., 2010; Wolber et al., 2012). Identification of new genes involved in progressive hearing loss and study of their pathological mechanisms is essential to broaden the understanding of auditory function and pathology. In this study, we report our investigations into the mechanisms underlying one form of progressive hearing loss found in mice with a synaptojanin2 (*Synj2*) mutation.

Synaptojanin2 is a phosphatidylinositol (PI) phosphatase characterized by three domains: an N-terminal similar to the Sac1 domain, a central PI 5-phosphatase domain, and a C-terminal proline-rich domain that can bind SH3 domain-containing proteins (Nemoto et al., 1997). In mouse, the *Synj2* gene has alternative splicing of the C-terminal giving 14 distinct transcripts of which 12 are translated into protein. Transcripts can derive from either of the two promoters in the 5′ UTR region, although the translation starts from the same start codon (Seet et al., 1998), or a promoter in intron 7 (Planchart, 2013).

The main function of both Synj2 and Synj1 is to remove the five-position phosphates from PI (3,4,5)-trisphosphate (PIP_3_) and PI 4,5-bisphosphate (PIP_2_). PIP_3_ dephosphorylation forms PtdIns (3,4)P_2_, while PIP_2_ dephosphorylation forms PtdIns(4)P. Both PIP_3_ and PIP_2_ are phospholipid components of the cell membrane and are involved in various functions; in particular, PIP_2_ is known to have a key role in exo- and endocytosis, ion channel modulation, and cell signaling regulation (Di Paolo and De Camilli, 2006). The synaptojanin family has been associated directly with membrane vesicle trafficking. Inhibition of *Synj2* by siRNA *in vitro* provoked a decrease in clathrin-coated pits and vesicles during endocytosis (Rusk et al., 2003). Synj1 has a critical role in endocytosis, as *Synj1* mutant mice die shortly after birth due to defects in synaptic vesicle recycling with an accumulation of PIP_2_ and clathrin-coated vesicles at nerve terminals (Cremona et al., 1999). Zebrafish with a *Synj1* mutation show basal blebs in sensory hair cells, a sign of imbalance between exo- and endocytosis, together with an increase in large coated vesicles and a reduction in numbers of tethered vesicles and reserve pool vesicles (Trapani et al., 2009).

In 2011, Manji et al. (2011) described the Mozart mutation, an ENU-induced single base change (c.1641T>A in exon 12; N538K) affecting the catalytic domain of the *Synj2* gene. An *in vitro* assay showed that the mutation inhibits the 5′ dephosphorylation activity of Synj2. Click-evoked auditory brainstem response (ABR) thresholds were normal in homozygous Mozart mutants at 4 weeks old but were severely affected by 8 weeks old. The progressive hearing loss was associated with stereocilia fusion of outer hair cells (OHC) and hair cell degeneration from as early as 2 weeks old starting in the basal turn of the cochlea, progressing with age to include inner hair cells (IHCs) and extending towards the middle-apical part of the cochlea. By 12 weeks old the mutants also exhibited reduced numbers of spiral ganglion cells. *Synj2* was reported to be expressed in hair cells (Manji et al., 2011). There were no behavioral signs of vestibular defects in these mutants, and peripheral nerve conductance recordings from the sciatic nerve show no systematic differences in mutants compared with control samples.

To investigate the mechanisms underlying progressive hearing loss, we have studied a different mutation of *Synj2* to look for any evidence of involvement of vesicle trafficking particularly affecting the synapses of sensory hair cells. ABR developed normally at first but started to decline between 3 and 4 weeks of age in *Synj2^tm1b^* mutants. At 6 weeks old, some evidence of OHC stereocilia fusion and degeneration was observed, but this was only seen in the extreme basal turn so cannot explain the raised ABR thresholds. We found no evidence of any defect in IHC exocytosis or endocytosis nor any sign of hair cell synaptic abnormalities, and endocochlear potential (EP) was normal. The mechanism underlying progressive hearing loss in these mutants remains elusive, but our findings of raised distortion product otoacoustic emission (DPOAE) thresholds and signs of OHC degeneration both suggest an OHC origin for the hearing loss.

## Materials and Methods

### Ethics Statement

Mouse studies were carried out following UK Home Office regulations and the UK Animals (Scientific Procedures) Act of 1986 (ASPA) under UK Home Office licenses, and the study was approved by the King’s College London, University of Sheffield and Wellcome Trust Sanger Institute Ethical Review Committees. Mice were culled using methods approved under these licenses to minimize any possibility of suffering.

### Generation of Mutant Mice

**Figure 1:**
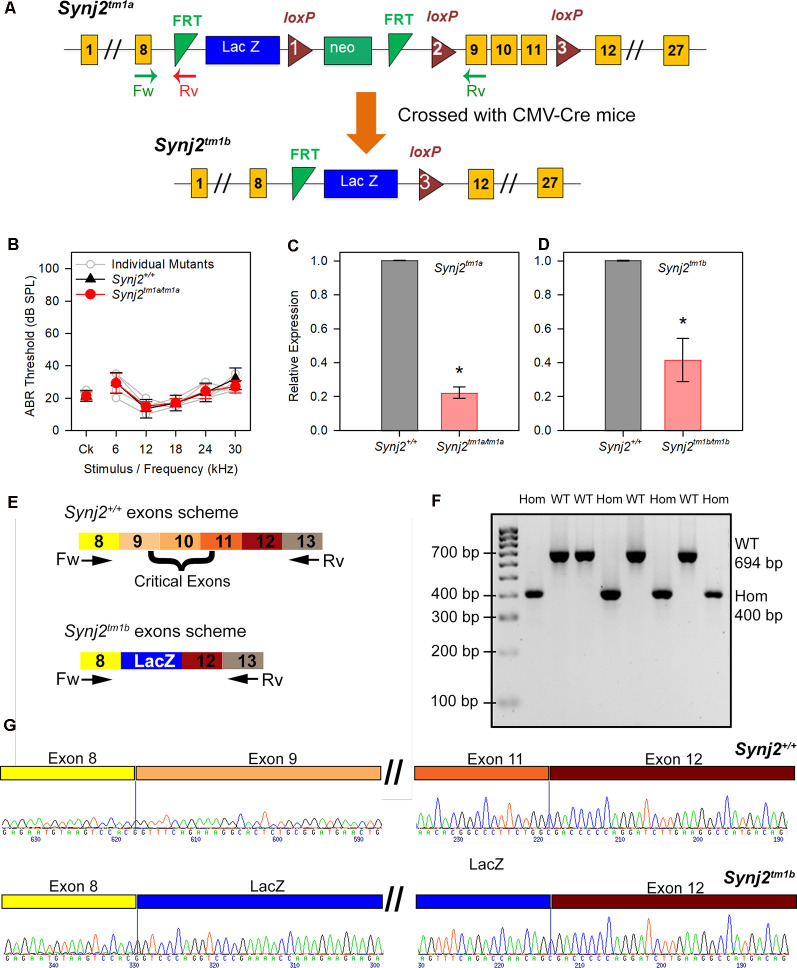
Generation of the Synaptojanin2 (*Synj2*; *Synj2^tm1a^*) and *Synj2^tm1b^* mutant mice. **(A)** Diagram showing the design of the *Synj2^tm1a^* and *Synj2^tm1b^* alleles. Yellow boxes show exons, green triangles show FRT sites, red triangles show loxP sites, blue and green boxes show the neomycin resistance and *LacZ* genes, arrows marked Fw and Rv indicate the locations of the genotyping primer sites. *Synj2^tm1a^* mutant mice were crossed to CMV-Cre mice to generate the *Synj2^tm1b^* mutant mice by Cre recombinase-mediated excision of the DNA between loxP sites 1 and 3, including deletion of exons 9, 10 and 11. **(B)** Auditory brainstem response (ABR) thresholds show no hearing impairment in *Synj2^tm1a/tm1a^* homozygous mice at 14 weeks old. *Synj2^+/+^* *n* = 7, black triangles; *Synj2^tm1a/tm1a^ n* = 7, red circles. Data plotted as mean ± standard deviation. **(C,D)** Quantitative RT-PCR shows knockdown of *Synj2* transcription in brain tissue from 4-week-old mice: *Synj2^tm1a/tm1a^* homozygotes have 22% of the normal level of transcribed mRNA and *Synj2^tm1b/tm1b^* homozygotes have 42%. *Hprt* was used as internal control and levels are normalized to wildtype (WT) levels (shown as 1.0 on the Y-axis). Three and four animals for each genotype were analyzed in panels **(C,D)** respectively. Data plotted as mean ± standard deviation. Mann–Whitney rank-sum test was performed: *Synj2^tm1a^* homozygous, *p = 0.05; *Synj2^tm1b^* homozygous, *p = 0.02. **(E)** Schematic representation of the WT *Synj2* transcript and the *Synj2^tm1b^* allele, where exons 9, 10, and 11 are excised and the LacZ gene is present. The two arrows correspond to the forward (Fw) primer on exon 8 and reverse (Rv) primer on exon 13 used for PCR amplification for genotyping. **(F)** Electrophoresis gel shows all the *Synj2^+/+^* (WT) samples have a band of 694 bp, while the band from the *Synj2^tm1b/tm1b^* (Hom) samples is around 400 bp. **(G)** Sanger sequencing results confirm the presence of exons 9, 10, and 11 in the *Synj2^+/+^* samples and their deletion in the *Synj2^tm1b/tm1b^* samples where the sequence of the *LacZ* gene is present between exons 8 and 12 instead. Four mice for each genotype were sequenced.

*Synj2^tm1a(EUCOMM)Wtsi^* (*Synj2^tm1a^*) mutant mice were generated at the Wellcome Trust Sanger Institute on a C57BL/6N genetic background (White et al., 2013). These mice carry a promoter-driven knockout-first allele, with a large cassette inserted in the intron before the targeted critical exons 9, 10, and 11 which interferes with transcription leading to knockdown of expression (Figure 1C). The inserted cassette contains the β-galactosidase/LacZ reporter gene (Skarnes et al., 2011; White et al., 2013). Further details can be found at www.mousephenotype.org. These mutant mice are available through the European Mouse Mutant Archive (EMMA). *Synj2^tm1b^* mutant mice were generated by crossing *Synj2^tm1a^* homozygotes with CMV-Cre-expressing mice (Figure 1A; Skarnes et al., 2011; White et al., 2013). Exposure to Cre recombinase led to recombination between LoxP sites, and founders for the *Synj2^tm1b^* colony were those that had recombination between loxP sites 1 and 3 (Figure 1A) and had undergone deletion of exons 9, 10 and 11. The CMV-Cre allele was bred out of the colony following recombination and the mice were maintained on a C57BL/6N genetic background.

### Genotyping

DNA was extracted from pinna skin and used as a template for short-range PCR using the forward primer 5′TAAGAGCGCCAGAACTTGGT3′ targeting exon 8 and the reverse primer 5′CATCGCTCAAACGACTCTCA3′ targeting exon 9 for the wild type (WT) allele, giving a band size of 385 bp from the WT allele but no PCR product from the *Synj2^tm1a^* allele as the primers were too far apart. The forward primer for the mutant allele was the same as that for the WT, and the reverse primer was 5′TCGTGGTATCGTTATGCGC3′, recognizing the first FRT site (Figure 1A) and giving a band size of 150 bp from the mutant allele but no PCR product from the WT allele as the FRT site was absent. The presence of the cassette was confirmed by testing for the neomycin resistance sequence using the forward primer 5′CAAGATGGATTGCACGCAGGTTCTC3′ and the reverse primer 5′GACGAGATCCTCGCCGTCGGGCATGCGCGCC3′. The *Synj2^tm1b^* allele was detected using the forward primer 5′CGGTCGCTACCATTACCAGT3′ recognizing the LacZ gene and the reverse primer 5′ACTGATGGCGAGCTCAGACC3′ which recognized the third LoxP site, and the deletion of the neomycin resistance gene sequence was confirmed using the primers used for detecting the *tm1a* allele, see above. Further detail of genotyping methods can be found elsewhere (White et al., 2013).

### β-Galactosidase Staining

Inner ears of 4-week-old *Synj2^tm1b^* mice were fixed in 4% paraformaldehyde for 2 h in rotation at room temperature (RT), washed twice with PBS for at least 30 min and decalcified in 0.1 M EDTA in rotation at RT until the bone was sufficiently soft (usually 2 or 3 days). The samples were washed for 30 min with a detergent solution (2 mM MgCl_2_; 0.02% NP-40; 0.01% sodium deoxycholate in PBS, pH 7.3). X-gal (Promega, cat.no. V394A) was added 1:50 to pre-warmed staining solution (5 mM K_3_Fe(CN)_6_ and 5 mM K_4_Fe(CN)_6_ in detergent solution), then the inner ears were stained at 37°C in the dark overnight. Following X-Gal staining, the samples were washed with PBS, dehydrated, and embedded in paraffin wax. The samples were sectioned at 8 μm, counterstained using Nuclear Fast Red (VWR, cat.no. 342094W) and mounted using Eukitt quick-hardening mounting medium (Sigma–Aldrich). The sections were imaged using a Zeiss Axioskop microscope connected to AxioCam camera and interfaced with Axiovision 3.0 software.

### Quantitative Reverse-Transcription PCR (qRT-PCR)

RNA was isolated from the whole brains of 4-week-old mice using TRI Reagent^®^ (Sigma–Aldrich) and the DNAse I kit (Sigma–Aldrich, cat.no. AMP-D1) was used to degrade any DNA residue in the samples, before the generation of cDNA using the Superscript II Reverse Transcriptase kit (Invitrogen, cat. no. 11904-018). To quantify the level of *Synj2* gene expression, cDNA was added to SSOFast Advanced Universal Probes Supermix (BioRad) and the *Synj2* Taqman^®^ probe (4351372, Life Technologies). Each sample was repeated in triplicate and the mean fold change between wild-type and homozygous mice replicate results were normalized to *Hprt* (Kang et al., 2013). The 2-ΔΔCT calculation (Livak and Schmittgen, 2001) was used to analyze the results and the Mann–Whitney rank-sum test was chosen for the statistical analysis.

### Sequencing

A new pair of primers were designed using Primer 3 upstream and downstream of the critical exons, recognizing exons 8 (TACACTGGGAAGACTTCGGC) and 13 (TCCACCATCTCCTCAAACCC) of the *Synj2* gene. The same cDNA used for the RT-qPCR was used as a template for PCR amplification. Before sequencing, they were incubated at 37°C for 30 min followed by 15 min at 80°C with ExoProStar (Illustra, cat. No. US77720V). Sanger sequencing was carried out by Source BioScience and the results were analyzed using Gap4.

### Inner Ear Clearing

Inner ears from 4-week-old mice were fixed with Bodian’s fixative (75% EtOH, 5% acetic acid, 5% formalin) for 2 h at 4°C in rotation (Pau et al., 2004). After washing with water, the samples were placed in 70% EtOH for 24 h at RT and then in 3% KOH for 5 days at RT with rotation, followed by clearing in glycerol, 70% EtOH and benzol (2:2:1) for 24 h. The specimens were stored in Glycerol: 70% EtOH (1:1) solution at 4°C and imaged using a Leica M216 microscope connected to the DFC490 Leica camera.

### Scanning Electron Microscopy

Cochlear samples from 6 weeks old *Synj2* homozygotes (*n* = 5) and littermate heterozygotes (*n* = 6) and WTs (*n* = 3) were fixed in 2.5% glutaraldehyde in 0.1 M sodium cacodylate buffer with 3 mM calcium chloride, dissected to expose the organ of Corti, then processed by a standard osmium tetroxide-thiocarbohydrazide (OTOTO) protocol (Hunter-Duvar, 1978). After dehydration, samples were subjected to critical point drying, mounted, and viewed using an FEI Quanta 200F scanning electron microscope (SEM). An overview of the cochlea was imaged to allow calculation of percentage distances along the cochlear duct to superimpose the frequency-place map (Müller et al., 2005), allowing subsequent imaging of consistent locations across different specimens. The numbers of hair bundles were quantified by manual counting in the following cochlear regions: 0–10% (of the distance along the cochlear duct from the hook) ≥60 kHz; 11–20% = 45–58 kHz; 21–30% = 34–44 kHz; 31–40% = 26–33 kHz; 41–50% = 20–25 kHz. Hair bundle survival was tested by one way ANOVA or Kruskal–Wallis one way ANOVA on Ranks followed by Dunn’s method *post hoc*, depending on the normality and equal variance tests.

### Synaptic Labeling and Confocal Imaging

Inner ears were fixed in 4% paraformaldehyde for 2 h and decalcified in EDTA overnight at RT. Following fine dissection, the organ of Corti was permeabilized in 5% Tween PBS for 40 min and incubated in a blocking solution (4.5 ml of 0.5% Triton X-100 in PBS and 0.5 ml of normal horse serum) for 2 h. The primary antibodies used overnight at RT were mouse anti-GluR2 (1:200, MAB397, Emd Millipore), rabbit anti-Ribeye (1:500, 192 103, Synaptic Systems), mouse anti-Ctbp2 (1:400, BD Transduction Laboratories 612044), and chicken anti-NF-H (1:800, Abcam ab4680). The samples were incubated for 45 min at RT with the secondary antibodies Donkey anti-mouse IgG Alexa Fluor594 (A21203, Thermo Fischer Scientific, Waltham, MA, USA); Goat anti-chicken IgG Alexa Fluor488 (A11039, Thermo Fischer Scientific); goat anti-rabbit IgG Alexa Fluor546 (A11035, Thermo Fischer Scientific); goat anti-mouse IgG2a Alexa Fluor488 (A21131, Thermo Fischer Scientific), and later were mounted using ProLong Gold mounting media with DAPI and stored at 4°C. Specimens were imaged using a Zeiss Imager 710 confocal microscope interfaced with ZEN 2010 software. The plan-APOCHROMAT 63× Oil DIC objective was used for all the images with 1.2 and 2.6 optical zooms for innervation and synapses staining respectively. Brightness and contrast were normalized for the dynamic range in all images.

The best-frequency areas were determined according to the mouse tonotopic cochlear map described by Müller et al. (2005). The number of ribbon synapses per IHC was quantified by counting manually the co-localized Ribeye and GluR2 puncta in the confocal maximum projection images and dividing it by the number of IHC nuclei (DAPI). An average of six IHCs per image was considered for the ribbon synapses counting using the cell- counter plugin in Fiji software. The normality of the data was determined using the Shapiro–Wilk and equal variance tests, and then the data were analyzed by the one-way ANOVA test.

### Auditory Brainstem Response (ABR) Measurements

Mice were anesthetized using 100 mg/kg Ketamine (Ketaset, Fort Dodge Animal Health) and 10 mg/kg Xylazine (Rompun, Bayer Animal Health) IP and positioned inside a sound-attenuating chamber (Industrial Acoustics Company Limited, Model 400-A) on a homeothermic blanket at 20 cm distance from the sound delivery speaker. Subcutaneous needle electrodes (NeuroDart; UNIMED, UK) were inserted on the vertex and overlying the left and right bullae (Ingham et al., 2011; Ingham, 2019). Mice needed for repeated ABR recordings were given 1 mg/kg atipamezole (Antisedan, Pfizer) IP to promote recovery from the anesthesia. Mice were tested at intervals starting at 4 weeks old, followed by 6, 8, 12, and 14 weeks old, and two separate cohorts were tested at 2 weeks and 3 weeks old. Free-field acoustic stimulation and recording of neural activity were controlled *via* a custom software application. Evoked responses picked up by the needle electrodes were amplified, digitized, and bandpass filtered between 300 and 3,000 Hz. To measure thresholds, we presented click stimuli (10 μs duration) and tone pips (5 ms duration, with a 1 ms onset and offset ramp) at a range of frequencies from 6 to 42 kHz over sound levels ranging from 0 to 95 dB SPL in 5 dB increments (Ingham et al., 2011, 2020), at a presentation rate of 42.6 stimuli per second. ABRs were recorded as an average of 256 presentations of each stimulus. Responses were stacked from low to high stimulus level to allow visual determination of threshold, defined as the lowest stimulus level which evoked a response waveform with characteristics consistent with features at higher stimulus levels, determined by visual inspection. Waveforms were analyzed and the latency and amplitude of ABR waves 1–4 were plotted as a function of the sound level above threshold (dB SL, sensation level).

The ABR thresholds were not normally distributed, therefore they were appropriately transformed before the analysis with a linear model with a compound symmetric covariance structure and restricted maximum likelihood estimation followed by Bonferroni *post hoc* test (Duricki et al., 2016). This test was preferred over the most common repeated-measures ANOVA because it enables all available data to be included in the analysis (Gueorguieva and Krystal, 2004; Krueger and Tian, 2004).

### Frequency Tuning Curves

Measurements of ABR wave 1 amplitude were used to generate frequency tuning curves (FTCs) using a forward masking stimulus paradigm (Ingham et al., 2020). Probe tones at 12 kHz, 18 kHz and 24 kHz (5 ms duration, 1 ms rise/fall time, presented at 20 dB above probe tone threshold) were presented with a 4 ms gap after a masker tone of variable frequency (in a ratio of 0.5, 0.7, 0.9, 0.95, 1.0, 1.05, 1.1, 1.3, 1.6, relative to the probe frequency), 10 ms duration, with a 1 ms rise/fall time, presented at levels ranging from 0 to 90 dB SPL in 10 dB steps. For each probe tone, the masked threshold was estimated for each masker frequency as the masker level that resulted in a 3 dB (50% magnitude) reduction in ABR wave 1 amplitude and plotted as a function of masker frequency to produce three FTCs for each mouse.

### Distortion Product Otoacoustic Emission (DPOAE) Measurements

The DPOAE is generated on the basilar membrane by OHCs following the presentation of two simultaneous long-lasting pure tones (f_1_ and f_2_) at a different frequency to the two input tones (Kemp, 1979). The two distinct tones were presented at a specific frequency ratio, f_2_/f_1_ = 1.20. The magnitude of the 2f_1_–f_2_ DPOAE component was extracted from a fast Fourier transform of the recorded microphone signal and plotted as a function of the f_2_ level.

Six weeks old mice were anesthetized with urethane (0.01 ml/g of a 20% solution, IP), a speculum was inserted into the ear canal and the detection probe microphone and sound delivery speakers were sealed into the speculum. The f_2_ tones were presented at 6, 12, 18, 24, and 30 kHz at increasing level 0–65 dB SPL in 5 dB steps, while the f_1_ was presented at 10 dB above the f_2_ (10–75 dB SPL). For each f_2_ frequency, the DPOAE threshold was determined at the lowest stimulus level (dB SPL) which evoked a 2f_1_–f_2_ DPOAE response that was two standard deviations above the mean noise floor. The DPOAE thresholds were statistically analyzed by the one way ANOVA or Kruskal–Wallis one way ANOVA on Ranks followed by Dunn’s method *post hoc*, depending on the normality and equal variance tests.

### Endocochlear Potential Recording

EP was measured in urethane-anesthetized mice (0.1 ml/10 g bodyweight of a 20% w/v solution) using 150 mM KCl-filled glass pipette microelectrodes, as described previously (Steel and Barkway, 1989; Chen et al., 2014a). EP was recorded as the potential difference between the tip of a glass microelectrode inserted into scala media *via* a fenestration in the cochlea basal turn lateral wall and a reference Ag-AgCl pellet electrode inserted under the skin of the dorsal surface of the neck. The positive EP was recorded as the steady-state positive potential obtained on the insertion of the electrode into the scala media. To measure the negative (anoxia) EP, the mouse was overdosed with an intraperitoneal injection of urethane and the resulting potential tracked for 30–60 min. Following the onset of hypoxia, EP began to decline, followed by a rapid fall to negative values as the mouse became anoxic. The negative EP was determined as the maximal negative potential recorded before the ionic balance within the inner ear started to drift back towards equilibrium and a zero EP.

### Single-Hair Cell Electrophysiology

Inner hair cells (IHCs) were studied in the acutely dissected cochlea from the apical-coil (6–12 kHz) and basal-coil regions (25–45 kHz) of adult mice. Cochleae were dissected in normal extracellular solution (in mM): 135 NaCl, 5.8 KCl, 1.3 CaCl_2_, 0.9 MgCl_2_, 0.7 NaH_2_PO_4_, 5.6 D-glucose, 10 Hepes-NaOH. Sodium pyruvate (2 mM), MEM amino acid solution (50×, without L-Glutamine), and MEM vitamin solution (100×) were added from concentrates (Thermo Fisher Scientific, UK). The pH was adjusted to 7.5 (308 mOsmol kg^−1^). The dissected cochleae were transferred to a microscope chamber, immobilized with a nylon mesh (Corns et al., 2018; Jeng et al., 2020) and continuously perfused with a peristaltic pump using the above extracellular solution. The organs of Corti were viewed using an upright microscope (Olympus BX51, Japan; Leica, DMLFS, Germany). Hair cells were observed with Nomarski Differential Interface Contrast (DIC) optics (×63 or ×60 water immersion objective) and ×15 eyepieces.

Patch-clamp recordings were performed using an Optopatch (Cairn Research Limited, UK) amplifier. Patch pipettes were made from soda glass capillaries with a typical resistance in the extracellular solution of 2–3 MΩ. To reduce the electrode capacitance, patch electrodes were coated with surf wax (Mr. Zoggs SexWax, Seattle, WA, USA).

Potassium currents were recorded at RT (~22°C) using an intracellular solution containing (in mM): 131 KCl, 3 MgCl_2_, 1 EGTA-KOH, 5 Na_2_ATP, 5 Hepes-KOH, 10 Na_2_-phosphocreatine (pH 7.3; osmolality ~296 mmol kg^−1^). Data acquisition was controlled by pClamp software using Digidata 1440A boards (Molecular Devices, San Jose, CA, USA). Recordings were low-pass filtered at 2.5 kHz (eight-pole Bessel), sampled at 5 kHz, and stored on a computer for off-line analysis (Origin2020, OriginLab, Northampton, MA, USA). Membrane potentials in voltage-clamp were corrected for the voltage drop across the uncompensated residual series resistance and a liquid junction potential (–4 mV).

Real-time changes in membrane capacitance (Δ*C*_m_) were performed at near body temperature (~35°C) and using 1.3 mM extracellular Ca^2+^ using the Optopatch amplifier as previously described (Johnson et al., 2013, 2017). Briefly, a 4 kHz sine wave of 13 mV RMS was applied to IHCs from −81 mV and was interrupted for the duration of the voltage step. The capacitance signal from the Optopatch was amplified (×50), filtered at 250 Hz, and sampled at 5 kHz. Δ*C*_m_ was measured by averaging the *C*_m_ trace over a 200 ms period following the voltage step and subtracting from pre-pulse baseline. For these experiments, the Cs-glutamate based intracellular solution was used (see above). Δ*C*_m_ and Ca^2+^ current recordings were performed in the presence of 30 mM TEA and 15 mM 4-AP (Fluka, Gillingham, UK) and Linopirdine (80 μM: Tocris, Bristol, UK) to block the K^+^ currents.

Statistical comparisons of means were made by the two-tailed *t*-test or, for multiple comparisons, analysis of variance, two-way ANOVA, followed by the Sidak post-test, was used means are quoted ±SEM, and *p* < 0.05 indicates statistical significance.

## Results

### Generation of a New Allele of *Synj2*

The *Synj2^tm1a (EUCOMM)Wtsi^(Synj2^tm1a^*) mutant mice were generated from targeted ES cells on a C57BL/6N genetic background (Skarnes et al., 2011; White et al., 2013). They carry a large intragenic insertion between exons 8 and 9 which inhibits transcription and translation of the gene (Figure 1A). When they were screened by ABR at 14 weeks old as part of the Sanger Institute Mouse Genetics Project (Ingham et al., 2019), they were found to have normal thresholds (Figure 1B). As this was unexpected, given the earlier report of hearing loss in the Mozart mutant mouse, we measured the level of knockdown of expression of *Synj2* using quantitative real-time PCR (RT-qPCR). RNA was extracted from brains of 4-week-old *Synj2^tm1a^* homozygotes and their littermate wild-type controls and was reverse transcribed to generate cDNA. *Hprt* was used as the internal control, and a *Synj2* TaqMan probe was chosen downstream of the inserted cassette, spanning exons 12 and 13. In the *Synj2^tm1a^* homozygotes, there was still 22% of the WT *Synj2* expression level (Figure 1C).

The normal ABR thresholds might have been due to this incomplete inactivation of the *Synj2* gene in the *Synj2^tm1a^* mutant mice. Therefore, exons 9, 10, and 11 were removed by crossing *Synj2^tm1a^* homozygotes with CMV-Cre-expressing mice to generate mice carrying the *Synj2^tm1b^* allele (Figure 1A; Skarnes et al., 2011; White et al., 2013). Exposure to Cre recombinase led to recombination between LoxP sites, and founders for the *Synj2^tm1b^* colony were selected from those that had recombination between the loxP sites 1 and 3 (Figure 1A) and had undergone deletion of exons 9, 10 and 11. The CMV-Cre allele was bred out of the colony following recombination and the mice were maintained on a C57BL/6N genetic background.

We then assessed the level of knockdown of *Synj2* expression in *Synj2^tm1b^* homozygotes by RT-qPCR as we did for the *Synj2^tm1a^* allele and surprisingly detected 42% of the normal level of mRNA (Figure 1D). However, amplification of the cDNA derived from *Synj2^tm1b^* homozygotes using primers designed to exon 8 and exon 13 produced a shorter band size as predicted (400 bp in mutants, 694 bp in WTs, Figures 1E,F) and sequencing confirmed that exons 9, 10 and 11 were deleted and replaced by the coding sequence of the *LacZ* gene (Figure 1G). The three exons deleted had several bases not divisible by three, leading to a predicted frameshift, so the *Synj2^tm1b^* allele is not expected to generate a functional protein. Furthermore, exon 11 encodes the first part of the phosphatase domain, so any protein produced from the *Synj2^tm1b^* allele is predicted to lack phosphatase activity. *Synj2* has 14 different transcripts and all but two of these include these three targeted exons.

*Synj2^tm1b^* homozygotes did not show any obvious vestibular defect, such as head bobbing or circling, at any of the ages studied.

### Synaptojanin2 Is Expressed in the Cochlea

**Figure 2:**
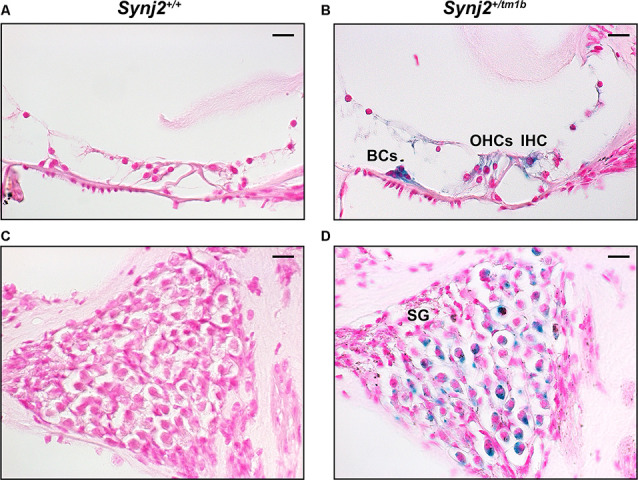
*Synj2* expression in the cochlea. *Synj2* is expressed in the outer and inner hair cells, some organs of Corti supporting cells (including the Boettcher cells shown here), and spiral ganglion at 4 weeks old, *n* = 2 for each genotype. **(A,C)** WT littermate controls were used to check the specificity of the X-gal staining. **(B,D)** X-gal staining (blue label) of sections of the inner ear of *Synj2^+/tm1b^* heterozygous mutant mice indicates the location of *Synaptojanin2* expression. Scale bars: 10 μm. OHCs, outer hair cells; IHC, inner hair cells; BCs, Boettcher cells; SG, spiral ganglion.

The inserted DNA cassette in the *Synj2^tm1b^* allele includes the *LacZ* gene, which encodes the β-galactosidase enzyme that can be used as a reporter of the usual expression pattern of the host gene. Using this approach in 4-week-old *Synj2^tm1b^* heterozygotes, the expression of *Synj2* was mainly detected in the IHCs and OHCs, in supporting cells such as Boettcher and Claudius’ cells, and in a subset of cells of the spiral ganglion (Figure 2). No signal was detected in the WT littermate controls (Figure 2).

### *Synj2^tm1b^* Homozygotes Show High-Frequency Progressive Hearing Impairment

**Figure 3:**
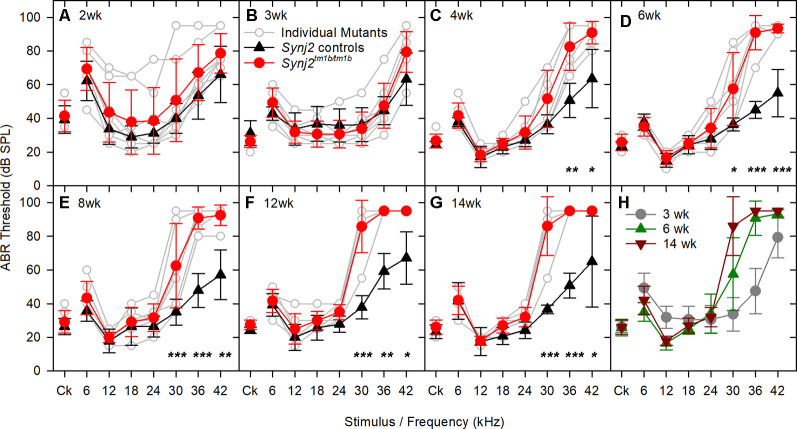
ABR thresholds decline with age in *Synj2^tm1b^* homozygotes. Mean ABR thresholds (± standard deviation) for clicks and tone pips are plotted for controls (black triangles) and homozygous *Synj2^tm1b^* mice (red circles). Pale gray lines show thresholds of individual homozygotes. The littermate control group included both WT and heterozygous mice as no difference was observed between them. The data were analyzed using separate linear models for each frequency with a compound symmetric covariance structure followed by the Bonferroni *post hoc* test. **(A,B)** ABR thresholds show no difference between controls (*n* = 20 in **A**, *n* = 12 in **B**) and mutants (*n* = 8 in both **A,B**). The variability in the 2 weeks old mice might be due to variability in the state of development of the hearing system, which is not uncommon at this early age. **(C–G)** The ABR thresholds at 30, 36, and 42 kHz increased progressively from 4 to 14 weeks in the mutants (*n* = 6) in comparison to the littermate controls (*n* = 7). Asterisks indicate *p < 0.05, **p ≤ 0.01 and ***p ≤ 0.001. **(H)** The ABR thresholds of the mutant mice are plotted for the age groups of 3, 6, and 14 weeks, showing a progressive rise of the thresholds at 30, 36, and 42 kHz.

Auditory function was investigated using ABR recording at different ages. *Synj2^tm1b^* mutant mice tested at 2 and 3 weeks old showed normal hearing sensitivity in comparison to littermate controls (Figures 3A,B) but showed progressively increased ABR thresholds at high frequencies from 4 weeks onwards (Figures 3C–H). Thresholds for 6–24 kHz and click stimuli remained close to normal up to 14 weeks old, while thresholds at 30, 36, and 42 kHz deteriorated (Figure 3H). These results suggest that *Synj2* is not required for the normal development of the auditory function, but it is needed for ongoing maintenance of sensitivity at high frequencies.

**Figure 4:**
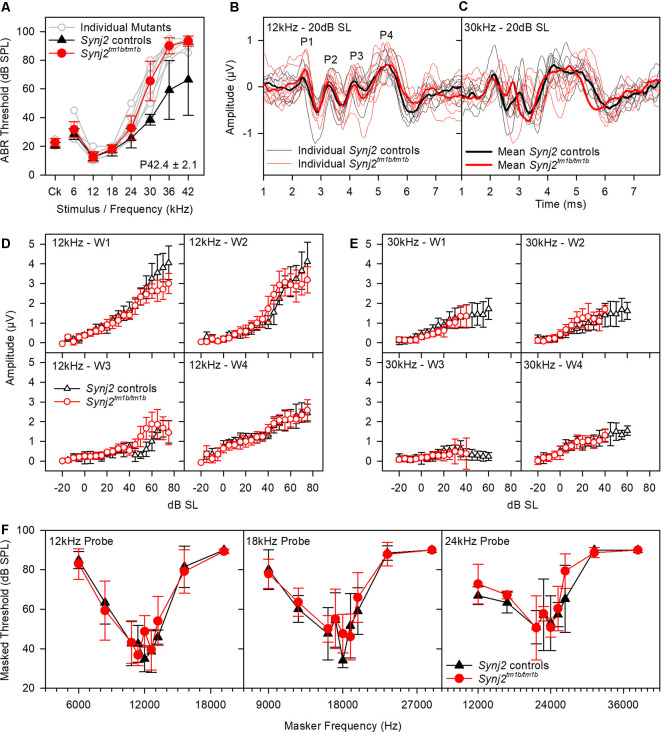
ABR waveform analysis and frequency tuning curves (FTCs). **(A)** ABR thresholds of the mice used in **(B–E)** are plotted for click stimuli and pure tones of 6–42 kHz. Thresholds of individual mutant mice are plotted in gray. Mean (± standard deviation) thresholds are plotted for controls (black triangles) and mutants (red circles). **(B,C)** Tone evoked ABR waveforms, recorded at 20 dB sensation level, are plotted for 12 kHz and 30 kHz stimuli. Responses from individual control mice are plotted as fine black lines and from individual mutant mice as fine red lines. The averaged waveform for each group is plotted as a thick line. **(B,C)** The mean amplitude (± standard deviation) of ABR waves 1–4 (W1, W2, W3, and W4) is plotted as a function of dB sensation level for control mice (black triangles) and mutant mice (red circles) for 12 kHz stimuli **(D)** and 30 kHz stimuli **(E)**. **(F)** FTC of control (black triangles; *n* = 5 WT, *n* = 2 heterozygote) and mutant (red circles, *n* = 9 homozygotes) mice, aged 6 weeks. Mean (± standard deviation) masked thresholds were comparable in control and mutant mice, using probe tones of 12, 18, and 24 kHz.

Reduced amplitude of ABR wave 1 has been suggested to indicate an IHC synaptic defect (Sergeyenko et al., 2013), so we analyzed the ABR waveforms recorded from 6-week-old mutants (Figures 4A–E). Average waveforms at a set stimulus level above threshold (sensation level, SL; see Figure 4A for thresholds) indicated no major systematic differences in shape between mutants and their littermate controls (Figures 4B,C). Amplitudes of waves 1–4 were plotted relative to increasing sound levels and showed no meaningful differences in mutants at either 12 kHz or 30 kHz, corresponding to frequencies with normal and raised thresholds respectively (Figures 4D,E).

**Figure 5:**
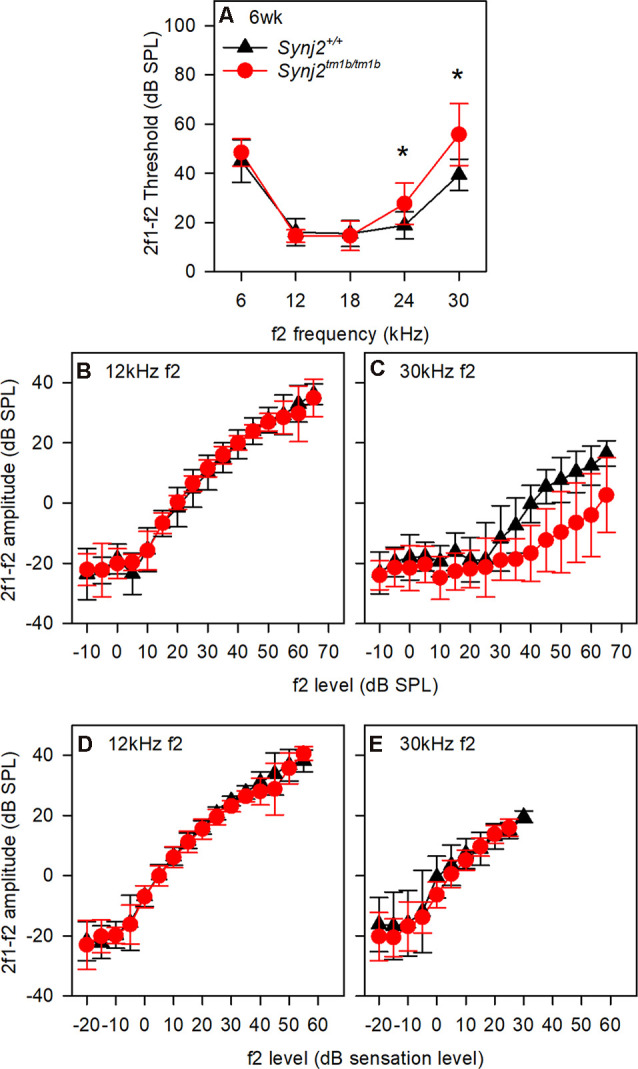
Distortion product otoacoustic emissions (DPOAE) show raised thresholds at high frequencies. **(A)** Mean (± standard deviation) 2f_1_–f_2_ thresholds for control (black triangles; WT, *n* = 9) and mutant (red circles; homozygote, *n* = 12) mice. f2 frequencies where significant threshold elevations were noted in mutant mice are indicated by asterisks (ANOVA, *p* < 0.05). **(B,C)** Mean (± standard deviation) 2f_1_–f_2_ amplitude is plotted as a function of dB SPL for control (black triangles) and mutant (red circles) mice, for f2 frequencies of 12 kHz **(B)** and 30 kHz **(C)**. **(D,E)** Mean (± standard deviation) 2f_1_–f_2_ amplitude is plotted as a function of dB sensation level SPL for control (black triangles) and mutant (red circles) mice, for f_2_ frequencies of 12 kHz **(D)** and 30 kHz **(E)**.

OHC function was assessed by recording 2f_1_–f_2_ DPOAEs in 6-week-old mice. Thresholds for detecting the DPOAE were slightly but significantly raised at high frequencies (Figure 5A) but the growth of amplitude of the emissions was similar in mutants and controls at f_2_ frequencies corresponding to normal thresholds (12 kHz, Figures 5B,D) and raised thresholds (30 kHz, Figures 5C,E) when plotted relative to sensation levels (Figures 5B–E). The increased thresholds suggested that OHC function was affected in the mutants.

OHC function is also thought to be important for normal frequency tuning (Dallos and Corey, 1991), so we used a forward masking paradigm to plot tuning curves based on the reduction of ABR wave 1 amplitude by masking stimuli of a range of other frequencies (Ingham et al., 2020). However, tuning curves for probe tones at 12, 18 and 24 kHz showed no significant differences between mutants and controls (Figure 4F). Thus, frequency tuning appears to be normal, at least at the tested frequencies.

### Endocochlear Potentials Are Normal in *Synj2^tm1b^* Mutants

**Figure 6:**
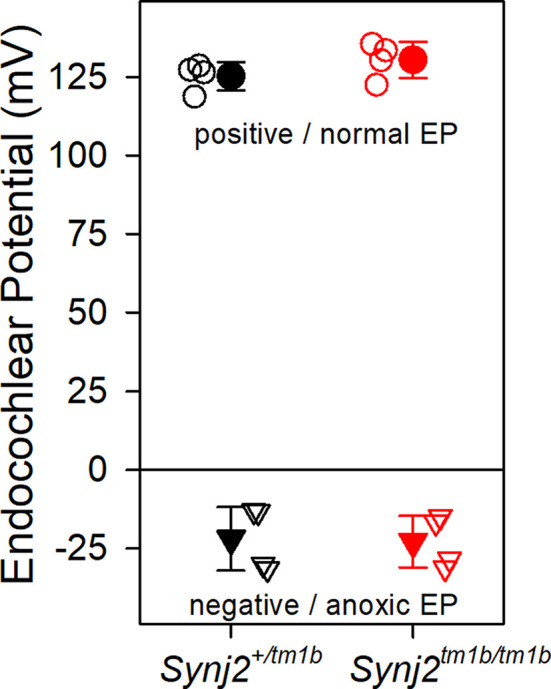
Endocochlear potentials (EP) are normal in mutants. Mean (± standard deviation) EP is plotted for heterozygote control mice (filled black, *n* = 4) and homozygote mutant mice (filled red, *n* = 4) aged 6 weeks. Empty symbols represent recordings from single mice. There was no difference in the normal positive EP (upper part of the graph shown by circles. ANOVA, *p* = 0.204), nor in the anoxia potential or negative EP (lower part of the graph shown by triangles. ANOVA, *p* = 0.898) between control and mutant mice. Both positive and negative EP were measured from the same mouse.

The EP was measured because a reduced EP would affect the function of both inner and OHC. Normal positive EPs were recorded in the *Synj2^tm1b^* mutants (Figure 6), indicating that the stria vascularis was functioning normally at 6 weeks old, an age when the mutants showed raised thresholds. When the cochlea becomes anoxic following cessation of the blood supply to the stria vascularis, the potential recorded in scala media drops rapidly to a negative level as positively-charged potassium ions leak out of scala media faster than anions can move in, resulting in a net negative potential, the anoxia potential (Bosher, 1979; Konishi, 1979; Steel and Bock, 1980). The leakage current is thought to flow mainly through the transduction channels of hair cells. In mutants where transduction channels are not functional or hair cells have degenerated, the anoxia potential does not reach such negative levels (Konishi, 1979; Steel and Bock, 1980). In the *Synj2^tm1b^* mutants, the anoxia potential reached the same negative level as in the control mice (Figure 6), indicating that most hair cells were present and their transduction channels were likely to be open to supporting the leakage current.

### *Synj2^tm1b^* Homozygotes Show Degeneration of OHC Bundles Only in the Extreme Basal Turn

**Figure 7:**
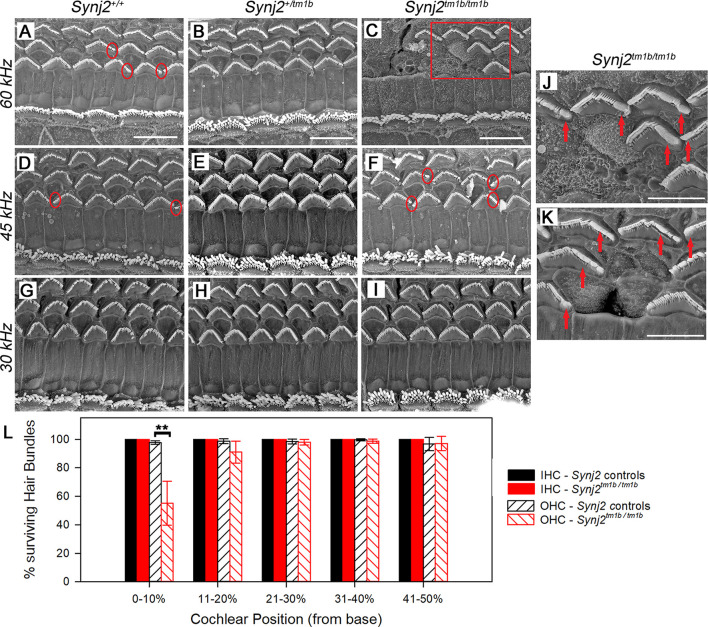
Scanning electron microscopy (SEM) of the organ of Corti in *Synj2^tm1b^* mutant mice. The littermate WTs, heterozygotes and homozygous mice at 6 weeks old were compared along the cochlear duct. Differences between genotypes were seen in the lower basal turns. Three basal regions of the cochlea are presented corresponding to best frequency regions of 60 kHz (**A–C**; 5–10% of the distance along the cochlear duct from the hook), 45 kHz (**D–F**; 15–20% distance) and 30 kHz (**G–I**; 35% distance). OHC degeneration was observed at the extreme base of the cochlea of *Synj2^tm1b/tm1b^* mice. All IHCs appeared normal in homozygotes compared with WTs and heterozygotes. **(A–C)** In the 60 kHz region, all OHCs were affected in *Synj2^tm1b/tm1b^* mice, showing fused stereocilia or missing bundles **(C)**, while in control mice, only a few OHCs have stereocilia fusion or are very rarely missing (as indicated by circles). **(D–F)** At the 45 kHz region, *Synj2^tm1b/tm1b^* mice **(F)** showed more frequent OHC stereocilia fusion (some examples in red circles) than controls **(D)**. **(G–I)** At the 30 kHz location, *Synj2^tm1b/tm1b^* mice **(I)** had a very similar appearance compared with *Synj2^+/tm1b^* or WTs **(G,H)**. **(J)** This shows an enlargement of the framed area in **(C)**. Red arrows point to examples of fused stereocilia. **(K)** Example of OHCs from another homozygote. Red arrows point to fused stereocilia and some hair bundles are missing. Scale bar: 10 μm in **(A–I)**; 5 μm in **(J,K)**. *Synj2^tm1b/tm1b^* *n* = 5; *Synj2^+/tm1b^* *n* = 6; *Synj2^+/+^* *n* = 3. **(L)** Quantification of hair bundle survival showed a significant reduction of OHC hair bundles at the location 0–10% of the distance along the cochlear duct from the hook (**p ≤ 0.01). No missing IHC hair bundles were observed in either controls or *Synj2^tm1b/tm1b^* mice. The five cochlear positions plotted correspond to the following frequency ranges: 0–10% ≥60 kHz; 11–20% = 45–58 kHz; 21–30% = 34–44 kHz; 31–40% = 26–33 kHz; 41–50% = 20–25 kHz. All data are shown as mean ± SD. 0–10% *p* = 0.006, controls *n* = 6 mice, *Synj2^tm1b/tm1b^* *n* = 5 mice; 11–20% *p* = 0.053, controls *n* = 9 mice, *Synj2^tm1b/tm1b^* *n* = 5 mice; 21–30% *p* = 0.633, controls *n* = 9 mice, *Synj2^tm1b/tm1b^* *n* = 5 mice; 31–40% *p* = 0.209, controls *n* = 7 mice, *Synj2^tm1b/tm1b^* *n* = 6 mice; 41–50% *p* = 0.938, controls *n* = 2 mice, *Synj2^tm1b/tm1b^* *n* = 3 mice. The littermate control group included both *Synj2^+/+^*and *Synj2^+/tm1b^* mice as no statistical difference was observed between them.

The gross structure of the inner ear of *Synj2^tm1b^* homozygotes appeared normal as observed in cleared samples. SEM was used next to view the surface of the organ of Corti of 6-week old *Synj2^tm1b^* homozygotes compared with their littermate heterozygous and WT controls (Figures 7A–L). The hair bundles of IHCs appeared normal along the whole length of the mutant cochlea. OHCs also had a normal appearance of their upper surface in the middle and apical turns. However, in the basal 20% of the cochlear duct, corresponding to best frequency responses of 45 kHz and above, some fusion of OHC stereocilia was observed at the ends of the V-shaped hair bundles (Figure 7F). This defect was progressively more severe moving down towards the base and there was a patchy loss of OHC hair bundles around the 60 kHz characteristic frequency region (0–10% of the distance from the base, Figures 7C,L). In the same regions, the control group showed some fused OHC stereocilia (Figure 7D), but it was much less frequent and less severe, and only a few sporadic OHC hair bundles were missing (Figures 7A,L). The organ of Corti appeared normal in *Synj2^tm1b^* mutants at regions corresponding to the impaired high frequencies (30, 36, and 42 kHz), as OHC degeneration started only at the extreme base of the cochlea (Figure 7L).

### Neurons and Ribbon Synapses of Hair Cells Show No Major Defects in *Synj2^tm1b^* Mutant Mice

**Figure 8:**
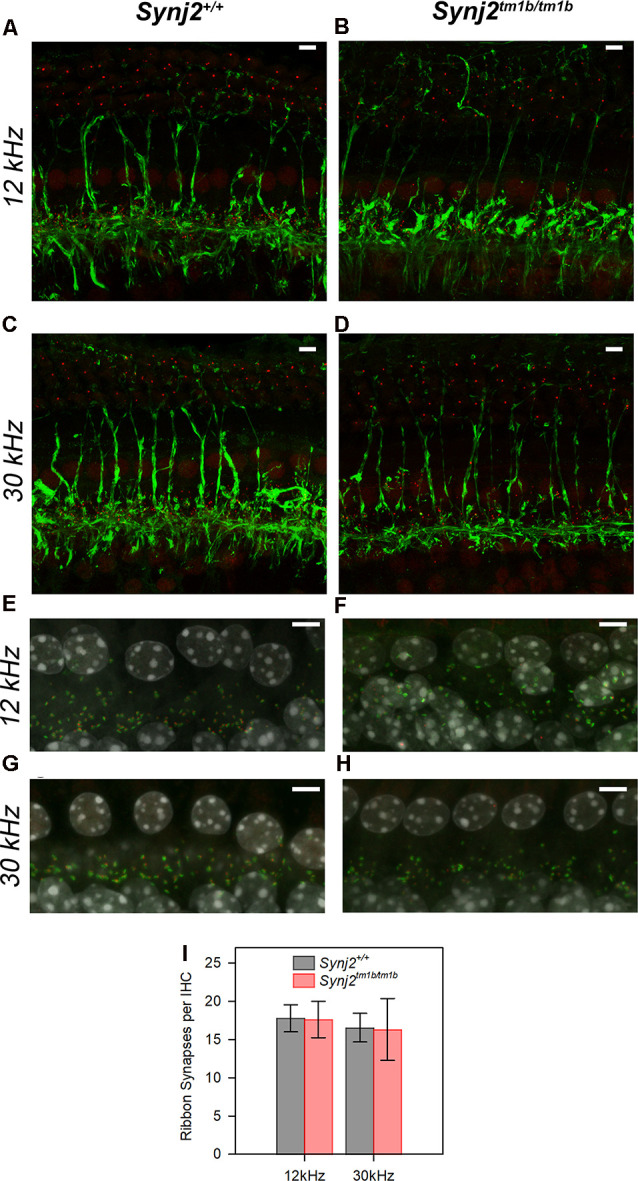
Innervation of the organ of Corti appears normal in mutants. **(A–D)**. The normal neuronal pattern in *Synj2^tm1b/tm1b^* mice. Maximum projection of stacks of confocal images of the whole-mount organ of Corti stained for neurofilament (green) and CtBP2 (red). The 12 k and 30 kHz best frequency regions of the cochlea were compared between the *Synj2^+/+^* mice **(A,C)** and the corresponding littermate *Synj2^tm1b/tm1b^* **(B,D)**. *Synj2^tm1b/tm1b^* *n* = 5; Controls *n* = 4 (three WT and one Heterozygote). Scale bar: 5 μm. **(E–H)** Confocal images of the whole-mount organ of Corti with Ribeye labeling of pre-synaptic ribbons in red, post-synaptic labeling of GluR2 in green, and the nuclei of cells in gray (DAPI). The cochlear regions corresponding to 12 and 30 kHz are compared between littermate *Synj2^+/+^* **(E,G)** and *Synj2^tm1b/tm1b^* **(F,H)** mice. Scale bar: 5 μm, *n* = 4 for both genotypes. **(I)** Quantification of ribbon synapses per IHC reveals no difference between *Synj2^tm1b/tm1b^* and control mice. Co-localized Ribeye and GluR2 puncta are counted and divided by the number of IHC nuclei in WT and *Synj2^tm1b/tm1b^* mice. All data are shown as mean ± SD and statistically analyzed by one-way ANOVA test, *n* = 4 for both genotypes. Number of synapses: WT 12 kHz 17.8 ± 1.8, 30 kHz 16.6 ± 1.9; *Synj2^tm1b/tm1b^* 12 kHz 17.6 ± 2.4 (*p* = 0.905); 30 kHz 16.3 ± 4.0 (*p* = 0.924).

As we found that *Synj2* was expressed in the spiral ganglion, and earlier reports of Synj2 function suggested a role in endocytosis (Rusk et al., 2003) implicating synaptic vesicle involvement, we examined the innervation of the organ of Corti in homozygotes and littermate WT aged 6 weeks. Unmyelinated nerve fibers of both afferent and efferent neurons were labeled with anti-neurofilament antibody (Kujawa and Liberman, 2009), but there were no obvious abnormalities in the overall arrangement of nerve fibers in the *Synj2^tm1b^* homozygotes (Figures 8A–D).

The integrity of the IHC synapses was studied using Ribeye and GluR2 antibody labeling. Ribeye is a marker for presynaptic ribbons and the GluR2 antibody labels the AMPA receptor subunit R2, which is part of the postsynaptic density. The normal juxtaposition of presynaptic ribbons (red) and postsynaptic terminals (green) was observed in the *Synj2^tm1b^* homozygous mice both in the 12 k and 30 kHz best frequency cochlear regions representing regions of normal and raised ABR thresholds respectively (Figures 8E–H). There was no significant difference in numbers of synapses at either 12 kHz (*p* = 0.905) or 30 kHz (*p* = 0.924) best frequency locations (Figure 8I).

### Synaptojanin2 Is Not Required for Exocytosis and Endocytosis in Adult IHCs

**Figure 9:**
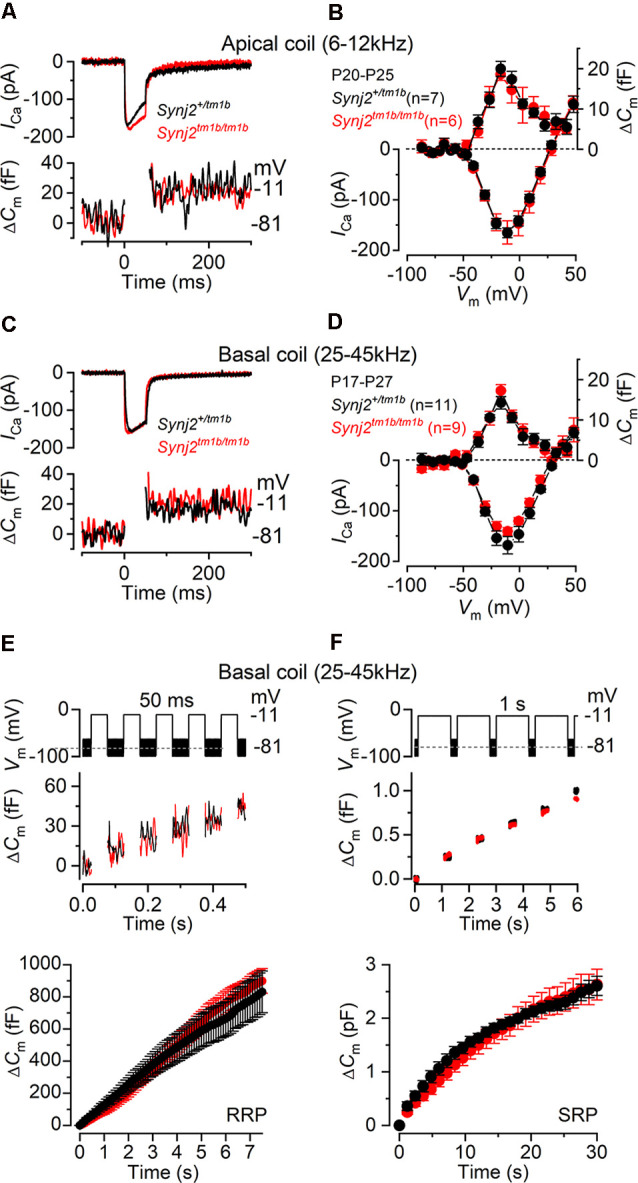
Exocytosis is normal in apical and basal IHCs from *Synj2* mice. **(A,B)** Calcium current (*I*_Ca_) and changes in membrane capacitance (*ΔC_m_*) recorded from control (*Synj2^+/tm1b^*, *n* = 7) and mutant (*Synj2^tm1b/tm1b^*, *n* = 6) apical-coil IHCs (6–12 kHz) of P20–P25 mice. Recordings were obtained in response to 50 ms voltage steps from −81 mV in 10 mV increments (in panel **A** only maximal responses at −11 mV are shown). **(C,D)** *I*_Ca_ and *ΔC_m_* recorded from 11 *Synj2^+/tm1b^* and nine *Synj2^tm1b/tm1b^* basal-coil IHCs (25–45 kHz) of P18–P23 mice using the protocol described above. **(E,F)** Δ*C*_m_ elicited using repetitive voltage steps to −11 mV of 50 ms and 1 s in duration to elicit the readily releasable pool (RRP) and secondary releasable pool (SRP), respectively. The inter-step-interval was either 50 ms **(E)** or 200 ms **(F)**. For clarity, only the first few steps are shown. The voltage protocol used is shown above the traces. Average cumulative Δ*C*_m_ values (bottom panels) obtained in response to the 50 ms and 1 s protocol, respectively, from 6 *Synj2^+/tm1b^* and 10 *Synj2^tm1b/tm1b^* IHCs. Error bars in all panels indicate SEM.

**Figure 10:**
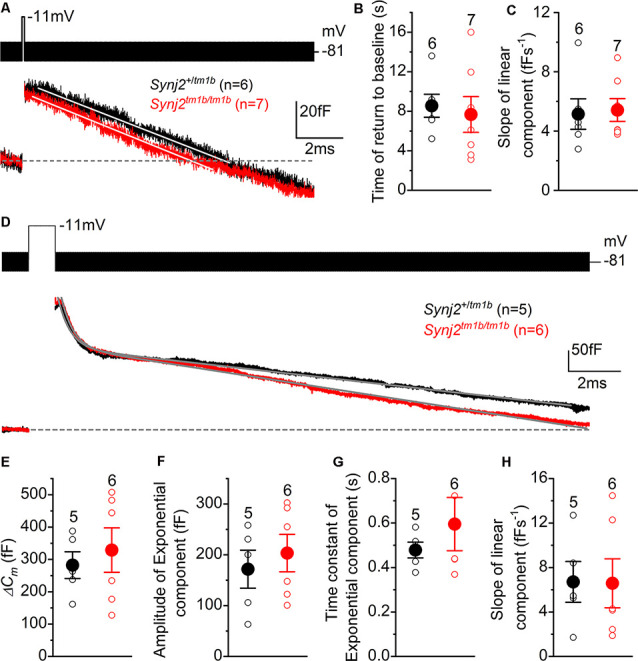
Endocytic responses in IHCs from *Synj2* mice. **(A)** Average depolarization-evoked exocytosis from basal coil IHCs of P17–P27 *Synj2* mice using mild stimuli (100 ms voltage step to −11 mV shown above the traces) able to mainly release the RRP. Continuous lines are the fits to the data using a linear function. **(B,C)** Average time required for the *C*_m_ to return to baseline **(B)** and average slope of the linear component of exocytosis **(C)** obtained from the fits to single IHC recordings (open symbols) to 100 ms voltage steps. **(D)** Average depolarization-evoked exocytosis from IHCs of P17–P27 *Synj2* mice using large stimuli (1 s voltage step to −11 mV) able to release the SRP. Continuous lines are the fits to the data using an exponential and linear function (see “Results” section). **(E)** Average Δ*C*_m_ elicited following a 1 s voltage step. **(F,G)** Average amplitudes **(F)** and time constants **(G)** of the exponential functions fitted to the data from single IHC recordings to 1 s stimulus. **(H)** The average slope of the linear component of exocytosis to 1 s voltage step.

The role of Synj2 of exocytosis and endocytosis was investigated from IHCs positioned in the apical-coil (6–12 kHz: P20–P25) and basal-coil regions (25–45 kHz: P17–P27) of the cochlea from adult mice (Figures 9, 10). The membrane capacitance of IHCs (*C*_m_), which is a measure of the total cell surface area, was found to be significantly larger in apical IHCs (10.1 ± 0.2 pF, 27 IHCs from both *Synj2^+/tm1b^* and *Synj2^tm1b/tm1b^* mice) than in basal cells (7.5 ± 0.2 pF, 48 IHCs, *p* ≤ 0.0001). *C_m_* was not significantly different when compared between *Synj2^+/tm1b^* and *Synj2^tm1b/tm1b^* mice at both cochlear regions (*p* < 0.05 for both 6–12 kHz and 25–45 kHz, one-way ANOVA, Tukey’s post-test).

Presynaptic function (i.e., exocytosis) in IHCs from adult *Synj2* mice was assessed by measuring the change in cell membrane capacitance (*ΔC_m_*) following cell depolarization (Figures 9A–F), which indicates vesicle fusion into the plasma membrane at presynaptic active zones (Johnson et al., 2013, 2017). The calcium current *I*_Ca_ and corresponding *ΔC_m_* were obtained by applying 50 ms voltage steps, which is known to recruit the readily releasable pool (RRP; Johnson et al., 2008). Adult IHCs from *Synj2^tm1b/tm1b^* mice had a comparable peak *I*_Ca_ to control littermates, *Synj2^+/tm1b^*, in both the apical (*p* = 0.9689, *t*-test, Figures 9A,B) and basal coil (*p* = 0.1927, Figures 9C,D) of the cochlea. The corresponding *ΔC_m_* was also not significantly different between the two genotypes in both apical (*p* = 0.5900) and basal (*p* = 0.1833) IHCs. Considering that the absence of *Synj2* affects the ABR thresholds above 24 kHz (Figure 3), we further investigated IHC presynaptic function by investigating the relative pool-refilling rates in the basal coil of the cochlea. The synaptic vesicle refilling rate was investigated by using repetitive depolarizing steps of either 50 ms or 1 s steps to −11 mV, which are normally used to investigate the depletion of the RRP and secondary releasable pool (SRP), respectively (Johnson et al., 2008). After repeated 50 ms steps, the cumulative *ΔC_m_* showed a near-linear increase in basal IHCs (Figure 9E), indicating that the RRP appears to be able to replenish after each step in both genotypes (*p* = 0.1148, Sidak’s post-test, two-way ANOVA). When a train of 1 s steps to −11 mV were applied, the cumulative *ΔC_m_* showed SRP release saturation that was also comparable between the two genotypes, (*p* = 0.2231, Figure 9F).

Considering that Synj2 has been shown to influence clathrin-coated pits and vesicles during endocytosis (Rusk et al., 2003), we sought to determine whether it has a similar role in IHCs. Mouse IHCs appear to exhibit two forms of endocytosis depending on the stimulus strength (Neef et al., 2014). While slow clathrin-mediated endocytosis (linear component) is used during mild stimulation that mainly recruits the RRP, bulk endocytosis (exponential component) is additionally used by IHCs to retrieve vesicles during the exocytosis of the SRP. Moreover, the dynamin inhibitor Dynasore has been shown to reduce exocytosis, indicating that clathrin-mediated endocytosis is important for the replenishment of synaptic vesicles at the IHC ribbon synapses (Duncker et al., 2013).

To investigate clathrin-mediated endocytosis in *Synj2* mice, mature P17–P27 IHCs from the basal coil cochlear region (25–45 kHz) were depolarized to −11 mV using a 100 ms voltage step, which is a mild stimulation causing a similar release of vesicles in both *Synj2^+/tm1b^* (38.5 ± 7.6 fF, *n* = 6) and *Synj2^tm1b/tm1b^* mice (36.6 ± 8.8 fF, *n* = 7, *p* = 0.8755, *t*-test, Figure 10A). Following the 100 ms voltage step, we followed *ΔC_m_* for 12 s to monitor vesicle reuptake. Both the time required for ΔC_m_ to return to baseline (Figure 10B) and the rate of vesicle uptake (Figure 10C) were not significantly different between *Synj2^+/tm1b^* and *Synj2^tm1b/tm1b^* mice (*p* = 0.7018 and *p* = 8337, respectively). Under our experimental condition (e.g., body temperature) the rate of membrane uptake (~5 fF s^−1^) was higher than that previously reported (~1.3 fF s^−1^: Neef et al., 2014). Finally, we found that after returning to baseline, the decline in *ΔC_m_* continued below the pre-stimulus level (Figure 10A); the reason for this “overshoot,” which was also described previously using perforated-patch recordings (Neef et al., 2014), is still unclear.

To investigate the bulk endocytosis (exponential component), IHCs were depolarized to −11 mV for 1 s to trigger the fusion of large numbers of synaptic vesicles, which was followed by a rapid exponential decrease in *ΔC_m_* and a slower linear decline (Figure 10D). This longer depolarization (1 s) induced a larger *ΔC_m_* that was not significantly different in IHCs from *Synj2^+/tm1b^* and *Synj2^tm1b/tm1b^* mice (*p* = 0.5956, Figure 10E). To quantify both the fast exponential and slow linear component of endocytosis, each recording was fitted with the following equation: *y = ce^−x/τ^* + *d−ax*, where *c* is the amplitude of the exponential component, *τ* is the time constant of the exponential decrease in *ΔC_m_*, *d* is the integration constant for the equation, and *a* is the rate of linear decrease in *ΔC_m_*. From the single fits, we found that the amplitude or rate constant of the exponential component of endocytosis, as well as that of the rate constant of the slower component, was not significantly different between the two genotypes (*p* = 0.5629, Figure 10F; *p* = 0.3777, Figure 10G; *p* = 0.9670, Figure 10H).

### Synaptojanin2 Is Not Required for the Functional Differentiation of Basal-Coil IHCs

**Figure 11:**
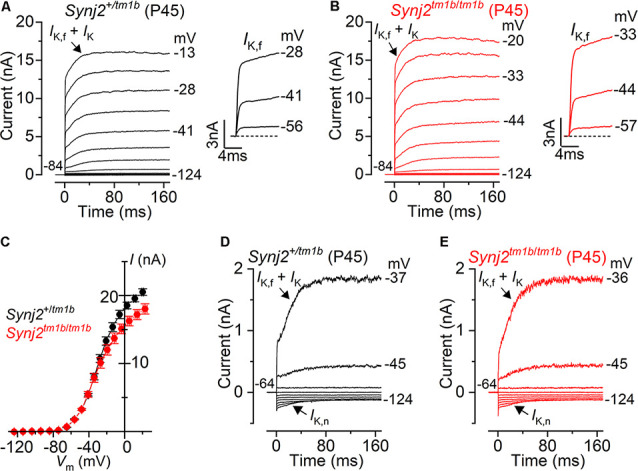
Current responses in mature IHCs from *Synj2* mice. **(A,B)** Potassium currents recorded from P45 control (*Synj2^+/tm1b^*: **A**) and mutant (*Synj2^tm1b/tm1b^*: **B**) basal-coil IHCs (25–45 kHz) of P45 mice, elicited by depolarizing voltage steps (10 mV nominal increments) from –124 mV to more depolarized values from the holding potential of –84 mV. The adult-type K^+^ current *I_K,f_* and *I_K,n_* are present in IHCs from both genotypes. **(C)** Steady-state current-voltage relationship for the total K^+^ current measured at 160 ms from eight *Synj2^+/tm1b^* and 7 *Synj2^tm1b/tm1b^* IHCs. **(D,E)** *I_K,n_* was more clearly visible when the K^+^ currents were recorded by using depolarizing voltage steps in 10 mV nominal increments from the holding potential of –64 mV.

The onset of adult-like characteristics in IHCs occurs at around P12 (Kros et al., 1998; Corns et al., 2014) with the expression of the negatively activating delayed rectifier K^+^ current (*I*_K,n_) and the fast activating large-conductance Ca^2+^ activated K^+^ current (*I*_K,f_; Marcotti et al., 2003). The K^+^ currents in adult IHCs from *Synj2* mice were recorded by applying a series of depolarizing voltage steps in 10 mV increments from –124 mV from the holding potential of –84 mV. We found that *I*_K,f_ and *I*_K,n_ were present in basal-coil IHCs (25–45 kHz) from both *Synj2^+/tm1b^* and *Synj2^tm1b/tm1b^* adult mice (P45: Figures 11A,B, respectively). The size of the outward K^+^ current measured at 160 ms and at 0 mV (Figure 11C) was not significantly different between *Synj2^+/tm1b^* (16.1 ± 1.5 nA, *n* = 8) and *Synj2^tm1b/tm1b^* (16.8 ± 0.8 nA, *n* = 7, P45, *p* = 0.6924, *t*-test). The size of the isolated *I*_K,f_, which was measured at 2.0 ms after stimulus onset at a membrane potential of −25 mV (Marcotti et al., 2003), was also comparable between *Synj2^+/tm1b^* (8.1 ± 0.8 nA, *n* = 8) and *Synj2^tm1b/tm1b^* (6.5 ± 0.4 nA, *n* = 7, P45, *p* = 0.1221). *I*_K,n_, was investigated by applying depolarizing voltage steps in 10 mV nominal increments from –124 mV to more depolarized values, starting from the holding potential of –64 mV (Figures 11D,E). The size of *I*_K,n_ was also similar between the two genotypes (*Synj2^+/tm1b^*: 134 ± 24 pA, *n* = 8; *Synj2^tm1b/tm1b^*: 118 ± 15 pA, *n* = 7, P45, *p* = 0.5930). The physiological consequence of the comparable voltage responses between the two genotypes was that adult *Synj2^tm1b/tm1b^* IHCs had a similar resting membrane potential (−73.9 ± 1.4 mV, *n* = 8) to that of *Synj2^+/tm1b^* cells (−75.4 ± 0.6 mV, *n* = 8, P45, *p* = 0.2679).

## Discussion

Here we report a new mutant allele of synaptojanin2, *Synj2^tm1b^*, which has a deletion of three exons, including exons 10 and 11 which encode part of the PI 5- phosphatase domain, and introduces a frameshift that should disrupt transcription and translation of the protein.

We initially examined the *Synj2^tm1a^* allele, which carries a large mutagenic insertion in the intron between exons 8 and 9 and was designed to act as a gene trap—interfering with transcription and knocking down the level of gene activity (Skarnes et al., 2011; White et al., 2013). The level of mRNA derived from this allele was reduced to around 20% of the levels in WT mice. However, the hearing was normal in *Synj2^tm1a^* homozygotes at 14 weeks old, suggesting that 20% of transcription is enough to allow normal auditory function. We then generated the *Synj2^tm1b^* allele by deleting exons 9, 10, and 11, and this led to progressive loss of auditory sensitivity. Surprisingly, mRNA levels were higher in this allele, around 40% of the normal WT level, despite the introduction of a frameshift which was expected to cause nonsense-mediated mRNA decay. This observation suggests that caution is needed in interpreting mRNA quantification as it may not fully reflect the impact of the mutation.

Homozygous *Synj2^tm1b^* mutant mice show normal development of auditory function followed by progressive hearing loss from 4 weeks old onwards. This finding indicates that synaptojanin2 is required for ongoing maintenance of hearing but not for the initial development of auditory sensitivity. As progressive hearing loss is so common in the human population, starting at any age, the human orthologue *SYNJ2* is a good candidate for involvement in human hearing loss. Indeed, two independent genome-wide association studies (GWAS) using more than 250,000 UK Biobank volunteers aged between 40 and 69 years, identified the *SYNJ2* gene as a significant risk locus for self-reported hearing difficulty (Kalra et al., 2019; Wells et al., 2019).

Our findings in the *Synj2^tm1b^* homozygotes corresponded broadly with those reported in the *Synj2^mozart^* mutant which has a missense mutation, Asn538Lys, affecting the catalytic domain of the protein. *Synj2^mozart^* mutants also showed a progressive increase in ABR thresholds to click stimuli and hair cell degeneration particularly affecting basal OHCs.

Our interest in the *Synj2* gene was stimulated by the reports of increased ABR thresholds in *Synj2^mozart^* mutants, and evidence that the encoded lipid phosphatase has a role in vesicle trafficking. However, we found no direct evidence of abnormal vesicle trafficking in *Synj2^tm1b^* mutant IHCs. What can we conclude about the initial site of the pathological process? As ABR thresholds at lower frequencies were normal, a defect in middle ear sound conduction is unlikely, and we saw no gross malformations of the inner ear in the mutants. EP were the same in mutants as in littermate controls, indicating normal stria vascularis function. Innervation of the organ of Corti appeared normal, including the dendrites crossing to the OHCs which are mostly efferent neurons, and the number of synapses at each IHC was the same in mutants as in controls, arguing against a primary neural defect despite the expression of *Synj2* in a subset of spiral ganglion cells. These observations point to the sensory hair cells as the likely site of the pathology. However, we saw no ultrastructural anomalies of IHCs by SEM and analysis of single IHC function using patch-clamp revealed no abnormality of their electrophysiological responses, and specifically no evidence of any deficiency in exocytosis or endocytosis. In contrast, at 6 weeks old we did see early signs of degeneration: OHC stereocilia fusion or complete OHC bundles were missing in the extreme basal turn and DPOAE thresholds were raised, implicating OHC involvement. OHC stereocilia defects and hair bundle loss were only seen in the extreme basal turn corresponding to best frequencies of 60 kHz or higher so hair cell degeneration *per se* cannot explain the raised ABR thresholds at 30, 36 and 42 kHz. The OHC stereocilia defects we observed in the basal 10% of the cochlear duct did not include major developmental defects such as disorganized stereocilia arrays, abnormal stereocilia shapes or sizes, giant stereocilia, or apparent dysregulation of stereocilia heights as reported in various other mutant animals, but instead was limited to the fusion of adjacent stereocilia and missing bundles. The fusion is similar (but less extensive) to the pathology reported in aging mouse OHCs (Bullen et al., 2019), the CD1 strain (Souchal et al., 2018), and *Pls1, Pjvk, Clic5*, and *Myo6* mutants among others (Self et al., 1999; Salles et al., 2014; Taylor et al., 2015; Kazmierczak et al., 2017). Anoxia EP was normal in the *Synj2* mutants which is consistent with most transduction channels (which carry the major part of the leakage current) being open. The normal resting potentials of single IHCs also indicate normal transduction in these cells. In summary, most potential sites for the initial lesion seem to have been ruled out by our findings in the *Synj2^tm1b^* mutants. The most likely cell type to be affected first is the OHC, and the lack of functional synaptojanin2 may affect OHC function before the onset of ultrastructural changes.

As synaptojanin2 is known to have a role in dephosphorylating PIP_3_ and PIP_2_, the impact of the *Synj2^tm1b^* mutation may be mediated by abnormal control of these phospholipids in the hair cell membranes. In the bullfrog saccular hair cell, PIP_2_ is predominantly located in stereocilia above the level of the taper and the basolateral hair cell membrane and is excluded from the apical surface and taper region (Hirono et al., 2004). Depletion of PIP_2_ in hair cells has been reported to decrease the peak transduction current, increase the open probability of transduction channels at rest, and decrease the rate of adaptation, consistent with a role for PIP_2_ in modulating transduction channel function (Hirono et al., 2004; Effertz et al., 2017). Ptprq is an alternative PI phosphatase also involved in deafness but with abnormal hair bundle development (Goodyear et al., 2003; Chen et al., 2014b) and it is located in PIP_2_-depleted regions (Oganesian et al., 2003), so maybe involved in the maintenance of a PIP_2_-free region in the hair bundles. The distribution of Synj2 protein within hair cells is not known, but the minimal impact of the *Synj2* mutation on hair bundle development suggests a different role to that of Ptprq.

PIP_2_ is known to regulate many different ion channels including key potassium channels important for hair cell functions such as BK and KCNQ channels (Hansen et al., 2011). It can act as a ligand, agonizing ion channels, changing their conformation, and hence influencing the cell’s resting membrane potential, as well as acting as a second messenger when cleaved and targeting specific proteins (including Synj2) to the plasma membrane (Hansen et al., 2011). KCNQ potassium channel isoforms, including KCNQ4, have been reported to be sensitive to PIP_2_ depletion (Suh et al., 2006; Leitner et al., 2011), although KCNQ4 channels show normal responses in the *Synj2* mutant IHCs we report here. PIP_2_ depletion also led to decreased Kv1 (*Kcna2*) channel activity in spiral ganglion neurons and slowed adaptation (Smith et al., 2015).

These studies on potassium channels and transduction channels have focused on the depletion of PIP_2_ while the *Synj2^tm1b^* mutant mice might be expected to have more PIP_2_ available. A fine balance of the PIP_2_ level might be important in inner ear homeostasis; abundance of this phospholipid might be as critical as its reduction for normal hearing function. Another membrane lipid, cholesterol, is important in prestin function in OHCs, and changes lead to reduced OHC motility and increased DPOAE thresholds (Rajagopalan et al., 2007). PIP_2_ cleavage has also been shown to lead to contractions of microvilli of *Drosophila* photoreceptors (Hardie and Franze, 2012). *Synj2^tm1b^* mutant mice have increased DPOAE thresholds so synaptojanin2 may also be required for normal OHC membrane function and motility.

A further possible role for Synj2 in normal hearing is suggested by the report of the phenotype of *Pip5k1c* mouse mutants (Rodriguez et al., 2012). *Pip5k1c* encodes a kinase that phosphorylates PI(4)P at the D5 position of the inositol ring generating PIP_2_. Homozygous mutants die shortly after birth with evidence of synaptic transmission defects. However, heterozygotes survive and show raised ABR thresholds at high frequencies, similar to the *Synj2* mutants, associated with reduced ATP-dependent Ca^2+^ signaling activity in the supporting cell network surrounding cochlear hair cells. We found *Synj2* expression in a subset of supporting cells, including Claudius cells and Boettcher’s cells. Rodriguez et al. (2012) found abnormal Ca^2+^ signaling only in the supporting cells on the modiolar side of IHCs, while Claudius and Boettcher’s cells are located on the lateral side of the OHCs, and the *Pip5k1c* mutation is expected to lead to reduced levels of PIP_2_ while the *Synj2* mutation most likely leads to increased PIP_2_ levels. Nonetheless, the similarities between the *Pip5k1c* and *Synj2* mutant phenotypes suggest that precise control of PIP_2_ signaling in supporting cells may play a role in normal hearing processes.

## Data Availability Statement

All datasets presented in this study are included in the article or are available on request from the authors.

## Ethics Statement

The animal study was reviewed and approved by King’s College London, University of Sheffield and Wellcome Trust Sanger Institute Ethical Review Committees.

## Author Contributions

EM performed RT-qPCR, sequencing, X-gal staining, immunofluorecence labeling, confocal imaging, post-acquisition image analysis, ABR at 2 and 3 weeks, and statistical analysis. NI carried out frequency tuning recording, ABR waveform analysis, DPOAEs, and EP measurements. OH and WM performed single hair-cell electrophysiological recordings and analysis. JP performed ABR at 4, 6, 8, 12 and 14 weeks and generated the *tm1b* allele. JC carried out SEM. KS and WM conceived the study, supervised the experiments, and the analysis and the interpretation of the data. KS, WM, and EM wrote the original draft. All authors contributed to the article and approved the submitted version.

## Conflict of Interest

The authors declare that the research was conducted in the absence of any commercial or financial relationships that could be construed as a potential conflict of interest.

## Footnotes

^1^http://www.actiononhearingloss.org.uk.

## References

[B1] Bosher S. K. (1979). The nature of the negative endocochlear potentials produced by anoxia and ethacrynic acid in the rat and guinea-pig. J. Physiol. 293, 329–345. 10.1113/jphysiol.1979.sp012892 41092 PMC1280716

[B2] Bullen A. Forge A. Wright A. Richardson G. P. Goodyear R. J. Taylor R. (2019). Ultrastructural defects in stereocilia and tectorial membrane in aging mouse and human cochleae. J. Neurosci. Res. 98, 1745–1763. 10.1002/jnr.24556 31762086

[B3] Chen J. Ingham N. Kelly J. Jadeja S. Goulding D. Pass J.. (2014a). Spinster homolog 2 (spns2) deficiency causes early onset progressive hearing loss. PLoS Genet. 10:e1004688. 10.1371/journal.pgen.1004688 25356849 PMC4214598

[B4] Chen J. Johnson S. L. Lewis M. A. Hilton J. M. Huma A. Marcotti W.. (2014b). A reduction in Ptprq associated with specific features of the deafness phenotype of the miR-96 mutant mouse diminuendo. Eur. J. Neurosci. 39, 744–756. 10.1111/ejn.12484 24446963 PMC4065360

[B5] Corns L. F. Bardhan T. Houston O. Olt J. Holley M. C. Masetto S. (2014). “Functional development of hair cells in the mammalian inner ear,” in Development of Auditory and Vestibular Systems, eds Romand R. Varela-Nieto I. (New York, NY: Academic Press), 155–188.

[B6] Corns L. F. Johnson S. L. Roberts T. Ranatunga K. M. Hendry A. Ceriani F.. (2018). Mechanotransduction is required for establishing and maintaining mature inner hair cells and regulating efferent innervation. Nat. Commun. 9:4015. 10.1038/s41467-018-06307-w 30275467 PMC6167318

[B7] Cremona O. Di Paolo G. Wenk M. R. Luthi A. Kim W. T. Takei K.. (1999). Essential role of phosphoinositide metabolism in synaptic vesicle recycling. Cell 99, 179–188. 10.1016/s0092-8674(00)81649-9 10535736

[B8] Dallos P. Corey M. E. (1991). The role of outer hair cell motility in cochlear tuning. Curr. Opin. Neurobiol. 1, 215–220. 10.1016/0959-4388(91)90081-h 1821184

[B9] Demeester K. Van Wieringen A. Hendrickx J. J. Topsakal V. Huyghe J. Fransen E.. (2010). Heritability of audiometric shape parameters and familial aggregation of presbycusis in an elderly flemish population. Hear. Res. 265, 1–10. 10.1016/j.heares.2010.03.007 20303401

[B10] Destefano A. L. Gates G. A. Heard-Costa N. Myers R. H. Baldwin C. T. (2003). Genomewide linkage analysis to presbycusis in the framingham heart study. Arch. Otolaryngol. Head Neck Surg. 129, 285–289. 10.1001/archotol.129.3.285 12622536

[B11] Di Paolo G. De Camilli P. (2006). Phosphoinositides in cell regulation and membrane dynamics. Nature 443, 651–657. 10.1038/nature05185 17035995

[B12] Duncker S. V. Franz C. Kuhn S. Schulte U. Campanelli D. Brandt N.. (2013). Otoferlin couples to clathrin-mediated endocytosis in mature cochlear inner hair cells. J. Neurosci. 33, 9508–9519. 10.1523/jneurosci.5689-12.2013 23719817 PMC3676539

[B13] Duricki D. A. Soleman S. Moon L. D. (2016). Analysis of longitudinal data from animals with missing values using SPSS. Nat. Protoc. 11, 1112–1129. 10.1038/nprot.2016.048 27196723 PMC5582138

[B14] Effertz T. Becker L. Peng A. W. Ricci A. J. (2017). Phosphoinositol-4,5-bisphosphate regulates auditory hair-cell mechanotransduction-channel pore properties and fast adaptation. J. Neurosci. 37, 11632–11646. 10.1523/jneurosci.1351-17.2017 29066559 PMC5707765

[B15] Gates G. A. Couropmitree N. N. Myers R. H. (1999). Genetic associations in age-related hearing thresholds. Arch. Otolaryngol. Head Neck Surg. 125, 654–659. 10.1001/archotol.125.6.654 10367922

[B16] Goodyear R. J. Legan P. K. Wright M. B. Marcotti W. Oganesian A. Coats S. A.. (2003). A receptor-like inositol lipid phosphatase is required for the maturation of developing cochlear hair bundles. J. Neurosci. 23, 9208–9219. 10.1523/jneurosci.23-27-09208.2003 14534255 PMC6740823

[B17] Gueorguieva R. Krystal J. H. (2004). Move over ANOVA: progress in analyzing repeated-measures data and its reflection in papers published in the archives of general psychiatry. Arch. Gen. Psychiatry 61, 310–317. 10.1001/archpsyc.61.3.310 14993119

[B18] Gurgel R. K. Ward P. D. Schwartz S. Norton M. C. Foster N. L. Tschanz J. T. (2014). Relationship of hearing loss and dementia: a prospective, population-based study. Otol. Neurotol. 35, 775–781. 10.1097/mao.0000000000000313 24662628 PMC4024067

[B19] Hansen S. B. Tao X. Mackinnon R. (2011). Structural basis of PIP2 activation of the classical inward rectifier K+ channel Kir2.2. Nature 477, 495–498. 10.1038/nature10370 21874019 PMC3324908

[B20] Hardie R. C. Franze K. (2012). Photomechanical responses in Drosophila photoreceptors. Science 338, 260–263. 10.1126/science.1222376 23066080

[B21] Hirono M. Denis C. S. Richardson G. P. Gillespie P. G. (2004). Hair cells require phosphatidylinositol 4,5-bisphosphate for mechanical transduction and adaptation. Neuron 44, 309–320. 10.1016/j.neuron.2004.09.020 15473969

[B22] Hunter-Duvar I. M. (1978). A technique for preparation of cochlear specimens for assessment with the scanning electron microscope. Acta Otolaryngol. Suppl. 351, 3–23. 10.3109/00016487809122718 352089

[B23] Ingham N. J. (2019). Evoked potential recordings of auditory brainstem activity in the mouse: an optimized method for the assessment of hearing function of mice. Bio Protoc. 9:e3447 10.21769/bioprotoc.3447 PMC7853958 33654942

[B24] Ingham N. J. Pearson S. Steel K. P. (2011). Using the auditory brainstem response (ABR) to determine sensitivity of hearing in mutant mice. Curr. Protoc. Mouse Biol. 1, 279–287. 10.1002/9780470942390.mo110059 26069055

[B25] Ingham N. J. Pearson S. A. Vancollie V. E. Rook V. Lewis M. A. Chen J.. (2019). Mouse screen reveals multiple new genes underlying mouse and human hearing loss. PLoS Biol. 17:e3000194. 10.1371/journal.pbio.3000194 30973865 PMC6459510

[B26] Ingham N. J. Rook V. Di Domenico F. James E. Lewis M. A. Girotto G.. (2020). Functional analysis of candidate genes from genome-wide association studies of hearing. Hear. Res. 387:107879. 10.1016/j.heares.2019.107879 31927188 PMC6996162

[B6100] Jeng J. Y. Ceriani F. Hendry A. Johnson S. L. Yen P. Simmons D. D.. (2020). Hair cell maturation is differentially regulated along the tonotopic axis of the mammalian cochlea. J. Physiol. 598, 151–170. 10.1113/JP279012 31661723 PMC6972525

[B1000] Johnson S. L. Forge A. Knipper M. Munkner S. Marcotti W. (2008). Tonotopic variation in the calcium dependence of neurotransmitter release and vesicle pool replenishment at mammalian auditory ribbon synapses. J. Neurosci. 28, 7670–7678. 10.1523/JNEUROSCI.0785-08.2008 18650343 PMC2516938

[B27] Johnson S. L. Kuhn S. Franz C. Ingham N. Furness D. N. Knipper M.. (2013). Presynaptic maturation in auditory hair cells requires a critical period of sensory-independent spiking activity. Proc. Natl. Acad. Sci. U S A 110, 8720–8725. 10.1073/pnas.1219578110 23650376 PMC3666720

[B28] Johnson S. L. Olt J. Cho S. Von Gersdorff H. Marcotti W. (2017). The coupling between Ca^2+^ channels and the exocytotic Ca^2+^ sensor at hair cell ribbon synapses varies tonotopically along the mature cochlea. J. Neurosci. 37, 2471–2484. 10.1523/jneurosci.2867-16.2017 28154149 PMC5354352

[B29] Kalra G. Milon B. Casella A. M. Song Y. Herb B. R. Rose K. (2019). Biological insights from multi-omic analysis of 31 genomic risk loci for adult hearing difficulty. BioRxiv. [Preprint]. 10.1101/562405 PMC7544108 32986727

[B30] Kang T. H. Park Y. Bader J. S. Friedmann T. (2013). The housekeeping gene hypoxanthine guanine phosphoribosyltransferase (HPRT) regulates multiple developmental and metabolic pathways of murine embryonic stem cell neuronal differentiation. PLoS One 8:e74967. 10.1371/journal.pone.0074967 24130677 PMC3794013

[B31] Kazmierczak M. Kazmierczak P. Peng A. W. Harris S. L. Shah P. Puel J. L.. (2017). Pejvakin, a candidate stereociliary rootlet protein, regulates hair cell function in a cell-autonomous manner. J. Neurosci. 37, 3447–3464. 10.1523/jneurosci.2711-16.2017 28209736 PMC5373128

[B32] Kemp D. T. (1979). Evidence of mechanical nonlinearity and frequency selective wave amplification in the cochlea. Arch. Otorhinolaryngol. 224, 37–45. 10.1007/bf00455222 485948

[B33] Konishi T. (1979). Some observations on negative endocochlear potential during anoxia. Acta Otolaryngol. 87, 506–516. 10.3109/00016487909126459 463522

[B34] Kros C. J. Ruppersberg J. P. Rusch A. (1998). Expression of a potassium current in inner hair cells during development of hearing in mice. Nature 394, 281–284. 10.1038/28401 9685158

[B35] Krueger C. Tian L. (2004). A comparison of the general linear mixed model and repeated measures ANOVA using a dataset with multiple missing data points. Biol. Res. Nurs. 6, 151–157. 10.1177/1099800404267682 15388912

[B36] Kujawa S. G. Liberman M. C. (2009). Adding insult to injury: cochlear nerve degeneration after “temporary” noise-induced hearing loss. J Neurosci 29, 14077–14085. 10.1523/JNEUROSCI.2845-09.2009 19906956 PMC2812055

[B37] Leitner M. G. Halaszovich C. R. Oliver D. (2011). Aminoglycosides inhibit KCNQ4 channels in cochlear outer hair cells *via* depletion of phosphatidylinositol(4,5)bisphosphate. Mol. Pharmacol. 79, 51–60. 10.1124/mol.110.068130 20935082

[B38] Lin F. R. Yaffe K. Xia J. Xue Q. L. Harris T. B. Purchase-Helzner E.. (2013). Hearing loss and cognitive decline in older adults. JAMA Intern. Med. 173, 293–299. 10.1001/jamainternmed.2013.1868 23337978 PMC3869227

[B39] Livak K. J. Schmittgen T. D. (2001). Analysis of relative gene expression data using real-time quantitative PCR and the 2(-delta delta C(T)) method. Methods 25, 402–408. 10.1006/meth.2001.1262 11846609

[B40] Manji S. S. Williams L. H. Miller K. A. Ooms L. M. Bahlo M. Mitchell C. A.. (2011). A mutation in synaptojanin 2 causes progressive hearing loss in the ENU-mutagenised mouse strain mozart. PLoS One 6:e17607. 10.1371/journal.pone.0017607 21423608 PMC3057978

[B41] Marcotti W. Johnson S. L. Holley M. C. Kros C. J. (2003). Developmental changes in the expression of potassium currents of embryonic, neonatal and mature mouse inner hair cells. J. Physiol. 548, 383–400. 10.1113/jphysiol.2002.034801 12588897 PMC2342842

[B42] Müller M. Von Hünerbein K. Hoidis S. Smolders J. W. (2005). A physiological place-frequency map of the cochlea in the CBA/J mouse. Hear. Res. 202, 63–73. 10.1016/j.heares.2004.08.011 15811700

[B43] Neef J. Jung S. Wong A. B. Reuter K. Pangrsic T. Chakrabarti R.. (2014). Modes and regulation of endocytic membrane retrieval in mouse auditory hair cells. J. Neurosci. 34, 705–716. 10.1523/jneurosci.3313-13.2014 24431429 PMC3891952

[B44] Nemoto Y. Arribas M. Haffner C. Decamilli P. (1997). Synaptojanin 2, a novel synaptojanin isoform with a distinct targeting domain and expression pattern. J. Biol. Chem. 272, 30817–30821. 10.1074/jbc.272.49.30817 9388224

[B45] Oganesian A. Poot M. Daum G. Coats S. A. Wright M. B. Seifert R. A.. (2003). Protein tyrosine phosphatase RQ is a phosphatidylinositol phosphatase that can regulate cell survival and proliferation. Proc. Natl. Acad. Sci. U S A 100, 7563–7568. 10.1073/pnas.1336511100 12802008 PMC164626

[B46] Pau H. Hawker K. Fuchs H. De Angelis M. H. Steel K. P. (2004). Characterization of a new mouse mutant, flouncer, with a balance defect and inner ear malformation. Otol. Neurotol. 25, 707–713. 10.1097/00129492-200409000-00010 15353999

[B47] Planchart A. (2013). Analysis of an intronic promoter within *Synj2*. Biochem. Biophys. Res. Commun. 440, 640–645. 10.1016/j.bbrc.2013.09.115 24103750 PMC5761313

[B48] Rajagopalan L. Greeson J. N. Xia A. Liu H. Sturm A. Raphael R. M.. (2007). Tuning of the outer hair cell motor by membrane cholesterol. J. Biol. Chem. 282, 36659–36670. 10.1074/jbc.M705078200 17933870 PMC2679373

[B49] Rodriguez L. Simeonato E. Scimemi P. Anselmi F. Cali B. Crispino G.. (2012). Reduced phosphatidylinositol 4,5-bisphosphate synthesis impairs inner ear Ca^2+^ signaling and high-frequency hearing acquisition. Proc. Natl. Acad. Sci. U S A 109, 14013–14018. 10.1073/pnas.1211869109 22891314 PMC3435166

[B50] Rusk N. Le P. U. Mariggio S. Guay G. Lurisci C. Nabi I. R.. (2003). Synaptojanin 2 functions at an early step of clathrin-mediated endocytosis. Curr. Biol. 13, 659–663. 10.1016/s0960-9822(03)00241-0 12699622

[B51] Salles F. T. Andrade L. R. Tanda S. Grati M. Plona K. L. Gagnon L. H.. (2014). CLIC5 stabilizes membrane-actin filament linkages at the base of hair cell stereocilia in a molecular complex with radixin, taperin and myosin VI. Cytoskeleton 71, 61–78. 10.1002/cm.21159 24285636 PMC4484851

[B52] Seet L. F. Cho S. Hessel A. Dumont D. J. (1998). Molecular cloning of multiple isoforms of synaptojanin 2 and assignment of the gene to mouse chromosome 17A2-3.1. Biochem. Biophys. Res. Commun. 247, 116–122. 10.1006/bbrc.1998.8564 9636665

[B53] Self T. Sobe T. Copeland N. G. Jenkins N. A. Avraham K. B. Steel K. P. (1999). Role of myosin VI in the differentiation of cochlear hair cells. Dev. Biol. 214, 331–341. 10.1006/dbio.1999.9424 10525338

[B54] Sergeyenko Y. Lall K. Liberman M. C. Kujawa S. G. (2013). Age-related cochlear synaptopathy: an early-onset contributor to auditory functional decline. J. Neurosci. 33, 13686–13694. 10.1523/jneurosci.1783-13.2013 23966690 PMC3755715

[B55] Skarnes W. C. Rosen B. West A. P. Koutsourakis M. Bushell W. Iyer V.. (2011). A conditional knockout resource for the genome-wide study of mouse gene function. Nature 474, 337–342. 10.1038/nature10163 21677750 PMC3572410

[B56] Smith K. E. Browne L. Selwood D. L. Mcalpine D. Jagger D. J. (2015). Phosphoinositide modulation of heteromeric Kv1 channels adjusts output of spiral ganglion neurons from hearing mice. J. Neurosci. 35, 11221–11232. 10.1523/jneurosci.0496-15.2015 26269632 PMC6605121

[B57] Souchal M. Labanca L. Alves Da Silva Carvalho S. Macedo De Resende L. Blavignac C. Avan P.. (2018). Transient abnormalities in masking tuning curve in early progressive hearing loss mouse model. Biomed. Res. Int. 2018:6280969. 10.1155/2018/6280969 29662891 PMC5832037

[B58] Steel K. P. Barkway C. (1989). Another role for melanocytes: their importance for normal stria vascularis development in the mammalian inner ear. Development 107, 453–463. 2612372 10.1242/dev.107.3.453

[B59] Steel K. P. Bock G. R. (1980). The nature of inherited deafness in deafness mice. Nature 288, 159–161. 10.1038/288159a0 7432512

[B60] Suh B.-C. Inoue T. Meyer T. Hille B. (2006). Rapid chemically induced changes of PtdIns(4,5)P2 gate KCNQ ion channels. Science 314, 1454–1457. 10.1126/science.1131163 16990515 PMC3579521

[B61] Taylor R. Bullen A. Johnson S. L. Grimm-Gunter E. M. Rivero F. Marcotti W.. (2015). Absence of plastin 1 causes abnormal maintenance of hair cell stereocilia and a moderate form of hearing loss in mice. Hum. Mol. Genet. 24, 37–49. 10.1093/hmg/ddu417 25124451 PMC4262491

[B62] Trapani J. G. Obholzer N. Mo W. Brockerhoff S. E. Nicolson T. (2009). Synaptojanin1 is required for temporal fidelity of synaptic transmission in hair cells. PLoS Genet. 5:e1000480. 10.1371/journal.pgen.1000480 19424431 PMC2673039

[B63] Wells H. R. R. Freidin M. B. Zainul Abidin F. N. Payton A. Dawes P. Munro K. J.. (2019). GWAS identifies 44 independent associated genomic loci for self-reported adult hearing difficulty in UK biobank. Am. J. Hum. Genet. 105, 788–802. 10.1016/j.ajhg.2019.09.008 31564434 PMC6817556

[B64] White J. K. Gerdin A. K. Karp N. A. Ryder E. Buljan M. Bussell J. N.. (2013). Genome-wide generation and systematic phenotyping of knockout mice reveals new roles for many genes. Cell 154, 452–464. 10.1016/j.cell.2013.06.022 23870131 PMC3717207

[B65] Wolber L. E. Steves C. J. Spector T. D. Williams F. M. (2012). Hearing ability with age in Northern European women: a new web-based approach to genetic studies. PLoS One 7:e35500. 10.1371/journal.pone.0035500 22558162 PMC3340381

